# Harnessing polarisation transfer to indazole and imidazole through signal amplification by reversible exchange to improve their NMR detectability

**DOI:** 10.1002/mrc.4607

**Published:** 2017-06-20

**Authors:** Marianna Fekete, Peter J. Rayner, Gary G. R. Green, Simon B. Duckett

**Affiliations:** ^1^ Centre for Hyperpolarization in Magnetic Resonance, Department of Chemistry University of York York YO10 5NY UK

**Keywords:** SABRE, indazole, imidazole, iridium catalyst

## Abstract

The signal amplification by reversible exchange (SABRE) approach has been used to hyperpolarise the substrates indazole and imidazole in the presence of the co‐ligand acetonitrile through the action of the precataysts [IrCl(COD)(IMes)] and [IrCl(COD)(SIMes)]. ^2^H‐labelled forms of these catalysts were also examined. Our comparison of the two precatalysts [IrCl(COD)(IMes)] and [IrCl(COD)(SIMes)], coupled with ^2^H labelling of the *N*‐heterocyclic carbene and associated relaxation and polarisation field variation studies, demonstrates the critical and collective role these parameters play in controlling the efficiency of signal amplification by reversible exchange. Ultimately, with imidazole, a 700‐fold^1^H signal gain per proton is produced at 400 MHz, whilst for indazole, a 90‐fold increase per proton is achieved. The co‐ligand acetonitrile proved to optimally exhibit a 190‐fold signal gain per proton in these measurements, with the associated studies revealing the importance the substrate plays in controlling this value. Copyright © 2017 The Authors. *Magnetic Resonance in Chemistry* published by John Wiley & Sons Ltd.

## Introduction

**Scheme 1:**
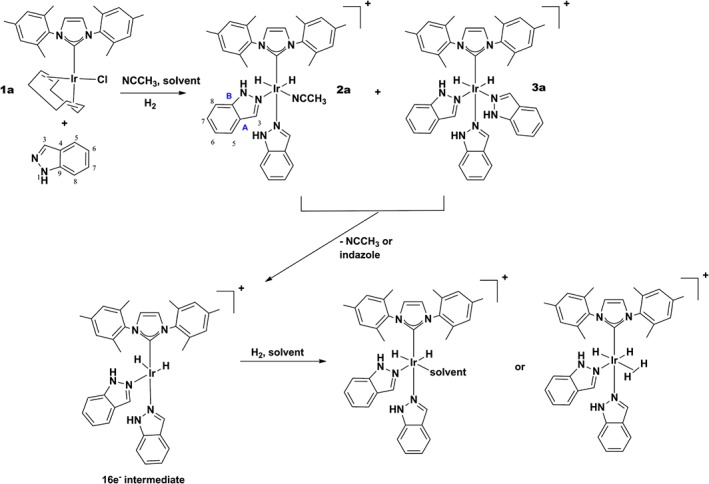
Formation of [Ir(H)_2_(ind)_2_(NCMe)(IMes)]Cl (**2a**) and [Ir(H)_2_(ind)_3_(IMes)]Cl (**3a**) via reaction of H_2_, acetonitrile and indazole (ind) with **1a**, labels as used in the text.

Hyperpolarisation methods are being used widely to improve the sensitivity of nuclear magnetic resonance (NMR) and magnetic resonance imaging (MRI) to substrate detection.^1^, ^2^ Signal amplification by reversible exchange (SABRE) is one such method where the nuclear spin order from *para*hydrogen (*p*‐H_2_) is used to sensitise substrate detection.^3^, ^4^, ^5^ The process of SABRE relies on breaking the magnetic symmetry of two protons that were originally located within a *p*‐H_2_ molecule, whilst retaining a spin–spin coupling between them in addition to introducing new couplings between them and the substrate to be hyperpolarised.^3^, ^5^ This is achieved by creating a SABRE catalyst that acts as a scaffold to bind both *p*‐H_2_ and the substrate such that it allows polarisation transfer through the resulting scalar coupling network. The process of SABRE is also affected by the magnetic field that is experienced by the catalyst during this process that is often called the polarisation transfer field (PTF).^6^ An active SABRE catalyst can break the symmetry of these two protons in one of two ways detailed in Scheme 1 for indazole, where the co‐ligand is acetonitrile and the precatalyst is [IrCl(COD)(IMes)] **1a**.^7^ As the original substrate molecule is reformed after ligand dissociation, there is no change in its chemical identity during this process, but it has now become a hyperpolarised (HP) species. SABRE catalysts based on *N*‐heterocyclic carbene (NHC) iridium complexes that contain 1,3‐bis(2,4,6‐trimethylphenyl)imidazol‐2‐ylidene (IMes) or 1,3‐bis(2,4,6‐trimethylphenyl)‐4,5‐dihydroimidazol‐2‐ylidene (SIMes) ligands are used in this study,^8^, ^9^ although studies on other templates and a range of substrates have been described in the literature.^6^, ^9^, ^10^ A number of theoretical approaches have been used to model the SABRE effect, of which the level‐anti‐crossing approach provides a readily understandable solution.^11^ Recently, the role of relaxation within the SABRE catalyst has also become recognised, and models are being developed that take this into account.^7^, ^12^ These studies have collectively confirmed that the time the substrate spends on the catalyst plays an import role in establishing the level of hyperpolarisation it gains. As consequence, the ligating power of the substrate, which is linked to the *p*K_a_ of the binding site, is important, as too strong an interaction can lead to low success by reducing overall exposure to *p*‐H_2_.

One of the drivers for the development of hyperpolarisation methods is the potential to collect *in vivo* data on a hyperpolarised agent that may ultimately prove to be diagnostic of health.^[10b]^ Another is the use of high‐sensitivity methods in analytical chemistry.[^10^, ^13^] Other routes to hyperpolarisation include dynamic nuclear polarisation,^2^ parahydrogen‐induced polarisation[^6^, ^14^] with substrate functionalisation and spin‐exchange optical pumping.[^10^, ^15^]

We have selected indazole (ind) and imidazole (im), for this study because this family of nitrogen‐containing heterocycles plays a role in a wide variety of biological processes.^16^ Additionally, the imidazole motif is also present in the amino acid histidine and the hormone histamine.^17^ Furthermore, antifungal agents such as flutrimazole and antibiotics such as metronidazole^12^ also contain this structural element.^17^, ^18^ Indazole derivatives therefore take an important place in healthcare as their biological activities include anti‐inflammatory, antimicrobial, anti‐HIV and anticancer roles.^16^, ^19^

Early reports on SABRE with indazole by Dücker *et al*. using the first generation of phosphine‐based SABRE catalysts^4^ were observed to produce a 2‐fold NMR signal enhancement.^[6a]^ A study on imidazole by Moreno *et al*.^[10d]^ using the second generation of carbene‐based SABRE catalysts^[6b]^ produced an improved response. Chekmenev *et al.* has also reported on the ^15^N hyperpolarisation of imidazole, with the supplementary information suggesting that a 100‐fold ^1^H signal enhancement is produced in H‐2 and 50‐fold gain in H‐4 and H‐5. Here, we describe a series of high‐field studies on polarisation transfer to both of these substrates where we detect their ^1^H and ^13^C NMR signals. We seek to improve on the degree of SABRE response by exploring the effect of catalyst structure and the use of a co‐ligand, in addition to varying the pH of the methanol solutions that are employed. It has recently been established that pH effects during SABRE can be substantial.[^10^, ^20^, ^21^]

The role for a co‐ligand during SABRE has been highlighted several times. In one manifestation, the use of a ^2^H‐labelled substrate allows the SABRE effect to be successfully focussed into the protons of a second substrate.^22^, ^23^ It has also proven possible to stabilise the active SABRE catalyst in order to successfully hyperpolarise weakly interacting substrates when substoichiometric amounts are available.^7^, ^24^, ^25^ Specifically, we show here that complex **2a** of Scheme 1 readily forms through the binding of two indazole ligands and one acetonitrile ligand. In SABRE catalysts of this type, the hydride ligands are therefore made chemically and magnetically inequivalent. The second form of catalyst, **3a**, contains three indazole ligands, and now, polarisation transfer is facilitated by magnetic inequivalence effects. The mechanisms of ligand exchange in these types of complex have been examined previously when the substrate is pyridine and underpin the SABRE effect.^7^, ^22^ These studies have enabled the hyperpolarisation of acetonitrile and reveal that it is possible to improve the pyridine response when CD_3_CN is used. More recently, the ^13^C and ^15^N hyperpolarisation of acetonitrile by SABRE has been considered.^22^, ^26^ Here, we show that, for type **2** complexes, the acetonitrile ligand is more labile than indazole and imidazole. The level of signal enhancement resulting from SABRE relates to the ligand exchange rate constants because polarisation transfer proceeds via the small J‐coupling that exists between the hydride ligand and polarisation acceptor.^2^ Understanding these effects is critical to optimisation of SABRE.^2^ It has also been suggested that because transfer is slow, relaxation by the SABRE catalyst can limit performance,^27^ and a simple and readily understandable model to assess this has been reported by Koptyug.^[11d]^ We therefore also report on the effects of catalyst deuteration on the level of SABRE by reference to appropriate isotopologues of IMes and SIMes, and by combining these approaches, we achieve high‐field signal gains in excess of 700‐fold. Our co‐ligand strategy also enables significant CH_3_CN hyperpolarisation to the achieved.

## Results and Discussion

### IrCl(NHC)(COD)‐derived signal amplification by reversible exchange of indazole: formation of [Ir(H)_2_(ind)_2_(NCCH_3_)(NHC)]Cl (**2**) and [Ir(H)_2_(ind)_3_(NHC)]Cl (**3**)

**Figure 1:**
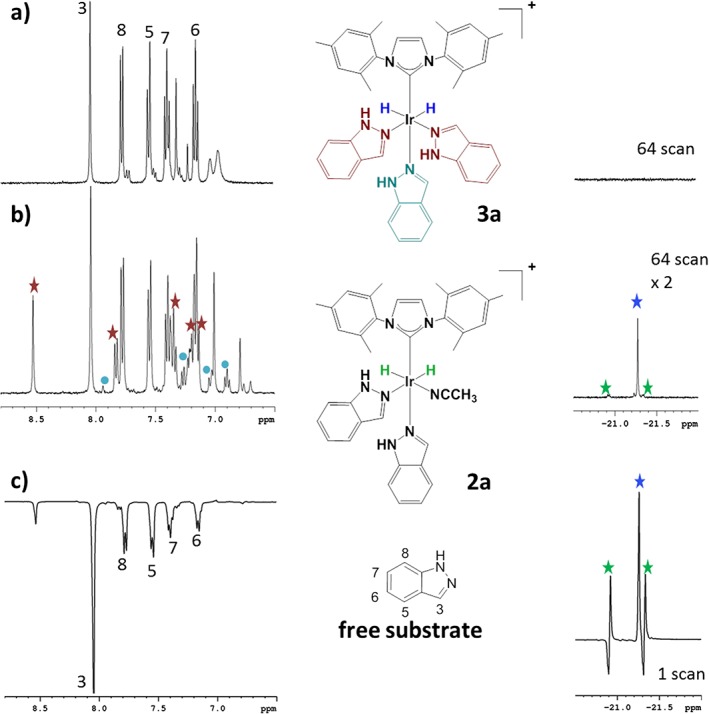
Plots (a), (b) and (c) show the aromatic and hydride regions of a series of ^1^H‐NMR spectra that were obtained in methanol‐*d*_4_ solution with **1a** and indazole.

[IrCl(COD)(IMes)] (**1a**) and [IrCl(COD)(SIMes) (**1b**) were found to react with indazole (4–10‐fold excess, relative to iridium) and acetonitrile (3‐fold excess) to form equilibrium mixtures of [Ir(H)_2_(ind)_2_(NCCH_3_)(IMes)]Cl (**2**) and [Ir(H)_2_(ind)_3_(IMes)]Cl (**3**)**.** These four complexes have been characterised by multinuclear NMR spectroscopy, the details of which can be found in the Supporting Information. In the case of **1a**, when the initial ligand ratio of indazole–acetonitrile is 10:3, products **2a** and **3a** exist in a 1:34 ratio, and hence, **3a** dominates. These complexes are diagnostically identified by the chemical shifts of their hydride ligands, which appear at *δ*_H_ −20.91 and −21.32 in **2a** and at *δ*_H_ −21.26 in **3a** respectively in MeOD at 298 K. The associated product ratio indicates a weaker Ir–NCMe bond, when compared with Ir–N_indazole_, and thus, **3a** is thermodynamically more stable than **2a**. In contrast, the analogous reaction with **1b** yields **2b** and **3b** in the ratio 1:50 under the same initial conditions. This indicates that the relative bond energies of the Ir–NC and Ir–N_indazole_ lie further apart when SIMes is the ancillary ligand as there is an even higher preference for **3b**. The corresponding hydride chemical shifts for **2b** are *δ*_H_ −20.95 and −21.28, whilst that for **3b** is *δ*_H_ −21.23. The hydride chemical shifts in the related complexes **2a** and **3a**, and **2b** and **3b**, are therefore very similar to one another and hence not strongly dependent on the identity of the NHC. Figure 1, panels (a), (b) and (c), shows the aromatic and hydride regions of a series of ^1^H‐NMR spectra in methanol‐*d*_4_ that were obtained with indazole. The NMR trace in panel (a) corresponds to one that was obtained before the addition of H_2_, and signals for H‐3 through H‐8 of indazole are indicated. The addition of H_2_ changes this response as **2a** and **3a** form with the new resonances for the coordinated indazole ligands being indicated with the * and ● labels (equatorial and axial ligands respectively, panel (b)). The hydride region of this NMR spectrum (right) reveals that **3a** dominates.

When this reaction is completed with *p*‐H_2_, and observed by ^1^H‐NMR spectroscopy, the hydride ligand signals of [Ir(H)_2_(ind)_2_(NCCH_3_)(NHC)]Cl (**2**) exhibit antiphase character due to the *para*hydrogen‐induced polarisation effect in the corresponding ^1^H‐NMR spectra (Fig. 1c, right). The average relative signal intensity gain for the polarised hydride resonances of **2a** is eight times larger than those of **2b** in comparable experiments (refer to Fig. 1c for a typical result). In addition, the equivalent hydride ligand signals of [Ir(H)_2_(ind)_3_(NHC)]Cl (**3**) both exhibit a weak in‐phase signal gain when they are first observed after sample transfer from low field, but this gain lies just 1.6‐fold in favour of **3a** over **2a**. The difference in **2a**:**2b** signal enhancement level is consistent with the fact that the observed rate of H_2_ loss at 298 K, as determined by exchange spectroscopy methods, from **2a** is 0.65 s^−1^, and just 0.08 s^−1^ for **3a**. Hence, there is more rapid *p*‐H_2_ introduction into **2a** and a larger signal gain results. These enhancement data therefore indicate that the presence of the acetonitrile facilitates more rapid IrH/H_2_ exchange in agreement with observations reported previously for the related products that form with pyridine.^7^

### Using an automated polariser

**Figure 2:**
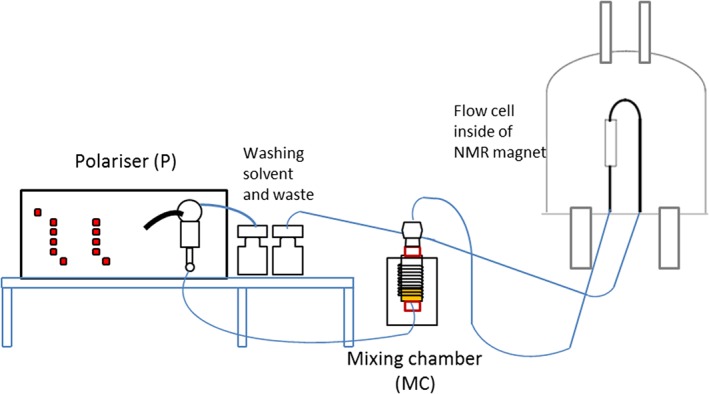
Schematic representation of the automated polariser used here to collect signal amplification by reversible exchange data.

A solution containing such a mixture of **2a** and **3a**, and free indazole, was subsequently probed for SABRE. These measurements were made in an automated flow apparatus that has been described previously.^[10a]^ This device, the Polariser, is represented in Fig. 2. It was designed to enable a solution that contains the catalyst and the hyperpolarisation target to be polarised using *p*‐H_2_ within a mixing chamber (MC) that is located in low field. The MC is surrounded by coil that can be used to generate a precise local magnetic field in the range −140 to +140 G and is located within a μ‐metal shield to screen the effect of the earth's field. For the flow measurements conducted in this study, a 3 ml volume of methanol‐*d*_4_ solution is typically employed that contains the iridium catalyst at a concentration of between 5 and 7 mM. It also contains 2–3 molar equivalents of the co‐ligand acetonitrile and 10 molar equivalents of the target substrate. Once a substrate is hyperpolarised, after bubbling *p*‐H_2_ through the solution for a predefined period, bubbling is stopped, and a 3‐s N_2_ purge activated. The solution then flows under nitrogen pressure into the NMR probe head for measurement in a process that takes 0.6 s, although a further delay of 0.1 s is added to allow the solution to settle before the NMR measurement is started. NMR measurement then proceeds in the usual way, although a receiver gain of 1 is typically employed to deal with the strongly enhanced signals we expect to detect. It takes therefore a total of 4.8 s to make a measurement after the SABRE step has been completed. During this time, the sample moves from low to high field and therefore experiences a range of magnetic environments and hence relaxation effects. These effects will act to reduce the level of detected SABRE and must play a larger role in shrinking the measured response of any rapidly relaxing signals. By employing this flow approach, the solution can ultimately be returned to the MC in order for it to be repolarised and the process started again. In this way, signal averaging, signal reproducibility and variable PTF plots can be constructed by repeated analysis of the same sample.

By using this equipment, we are able to precisely vary a number of parameters that control the SABRE effect in order to optimise them. When the initial concentration of **1a** in methanol‐*d*_4_ was 6.5 mM, and 49.4 mM of indazole and 17.0 mM of acetonitrile were introduced, strong SABRE enhancements were visible in the NMR signals of both these reagents. The maximum proton signal enhancement for the five non‐exchangeable protons of indazole proved to total 115‐fold that equates to a signal gain of ~20 per proton and resulted when the sample had been exposed to a 70 G magnetic field in conjunction with 20 s of exposure to *p*‐H_2_ (Supporting Information, Fig. S1). The corresponding acetonitrile proton signal was observed to achieve a maximum enhancement of 145‐fold after transfer at 80 G that equates to an ~50‐fold gain per proton (Supporting Information, Fig. S2). Previously, such a high level of polarisation transfer into acetonitrile has only been seen when a deuterated ligand scaffold is employed in conjunction with the co‐ligand pyridine.^7^ This study therefore reveals the importance of the co‐ligand in controlling the level of polarisation transfer into a weakly bound ligand, in this case acetonitrile. We note that Tessari et al. further developed the co‐ligand approach to enable analyte quantification at low loadings.^28^ Additionally, the efficiency of the SABRE effect is PTF dependant as a consequence of the matching conditions that must be met between the chemical shift and coupling values within the catalyst.^2^, ^29^ It is therefore usual to quote optimal PTF values when reporting data in a similar way to quoting absorption maxima in UV spectroscopy.

**Scheme 2:**
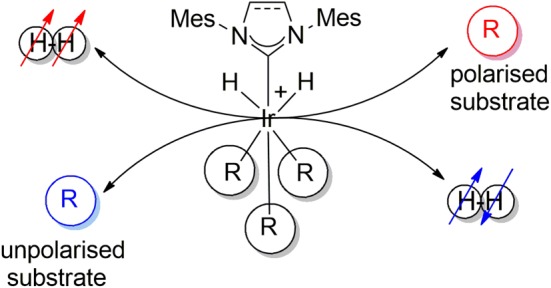
Conceptual representation of the signal amplification by reversible exchange process that achieves the catalytic hyperpolarisation of a substrate via polarisation transfer within an iridium catalyst from a pair of protons that were previously located in a molecule of *p*‐H_2_.

When a 6.6 mM solution of **1b** was examined in the presence of similar 10 and 3‐fold ligand excesses respectively, SABRE was again observed. Now, however, the total indazole proton signal enhancement within the five sites increased to 234‐fold (~47 per proton) after transfer at a PTF of 70 G and a 20‐s *p*‐H_2_ exposure time. The corresponding acetonitrile signal gain was 266‐fold at 80 G. Hence, the catalyst derived from **1b** exhibits superior performance to that derived from **1a**. This observation is in agreement with the fact that the effective rate of build‐up of indazole in solution, via dissociation from dominant **3a** and **3b**, at 298 K is 0.26 and 0.64 s^−1^ respectively. Scheme 2 illustrates the SABRE process in a conceptual form. The effective rate of build‐up of free substrate in solution used here is defined as the observed rate of magnetisation transfer from the H‐3 resonance of **3** into the corresponding signal for free indazole and has units of per second (refer to Supporting Information).

We also undertook a series of control measurements under analogous conditions without acetonitrile. The corresponding H‐3 signal of indazole was observed to yield a 330‐fold signal again under these conditions where **3a** is the catalyst after transfer at 60 G.

### Signal amplification by reversible exchange hyperpolarisation of residual CHD_2_OD and CD_3_OH

**Figure 3:**
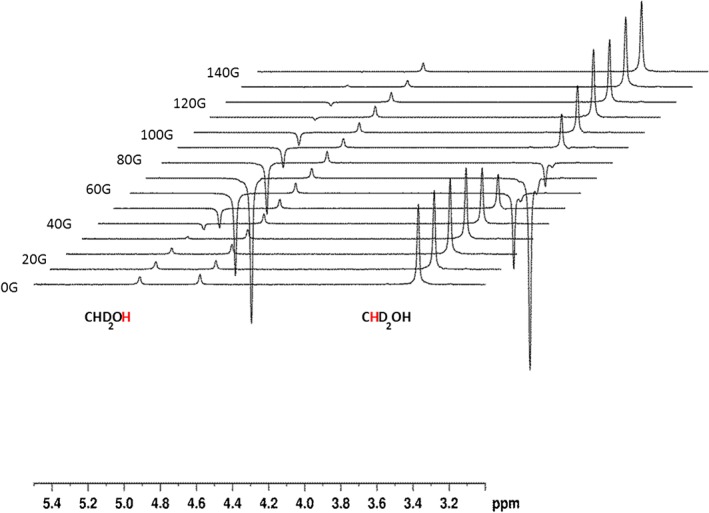
Series of ^1^H‐NMR spectra showing the signals for hyperpolarised CD_3_OH and CHD_2_OD that result from signal amplification by reversible exchange as a function of the polarisation transfer field. These data were collected using a methanol‐*d*_4_ solution that contained a 10‐fold excess of indazole and a 3‐fold excess of acetonitrile relative to **1a**.

Mechanistically, the loss of indazole from **3** and acetonitrile from **2** will lead to a common 16‐electron intermediate, [Ir(H)_2_(ind)_2_(NHC)]Cl, alongside hyperpolarised indazole and acetonitrile respectively (Scheme 1). Both H_2_, indazole or methanol can then coordinate to this intermediate, with *p*‐H_2_ binding providing the route by which the cycling of *para*hydrogen is achieved that underpins SABRE. As methanol is present in far larger excess than either indazole or acetonitrile, its binding is assured, and single‐spin hyperpolarisation is consequently observed in these experiments in the residual CHD_2_OD and CD_3_OH solvent signals as detailed in Fig. 3. For systems derived from **1a**, transfer into the solvent is most readily evident after transfer at 70 G, with both of the residual solvent signals showing a phase change, from absorption to emission, on moving between transfer fields of 50 and 60 G. The maximum signal enhancement seen for these signals, relative to those seen under thermal conditions, was 2.6‐fold for the CHD_2_OD peak and 3.0‐fold for the CD_3_OH peak. This small level of signal gain results from the low proportion of ^1^H‐labelled species in CD_3_OD, which means that most of the methanol binding to the associated 16‐electron intermediate [Ir(H)_2_(ind)_2_(NHC)]Cl forms [Ir(H)_2_(ind)_2_(CD_3_OD)(NHC)]Cl that will be unproductive for methanol‐SABRE. Furthermore, the concentration of [Ir(H)_2_(ind)_2_(methanol)(NHC)]Cl must also be low as this species is not detected in these NMR spectra.

**Figure 4:**
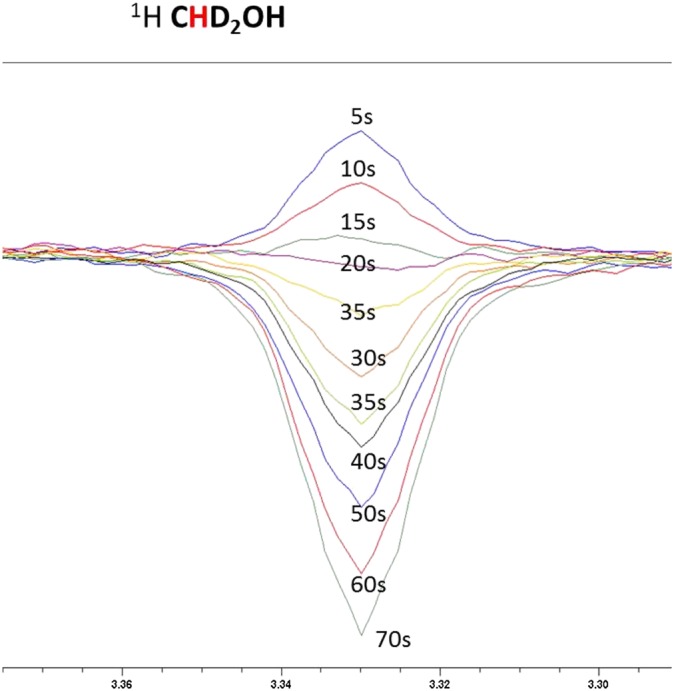
Plot of the *p*‐H_2_ bubbling time *versus* the level of CHD_2_OD signal gain. The change in signal phase results from the combination of a positive thermal signal and a negative hyperpolarised signal for the CHD_2_OD resonance.

Under SABRE, the level of signal enhancement builds as the exposure time to *p*‐H_2_ increases prior to reaching a relaxation‐controlled maximum.^22^ This is commonly referred to as the bubbling time when using the automated system described earlier, and it might be expected that a signal builds up in intensity before reaching a plateau.^[10a]^ The low efficiency of the methanol CHD_2_ enhancement allows this effect to be visualised, as detailed in Fig. 4 that shows a change in signal phase as the bubbling time is increased; the SABRE effect creates a negative signal that eventually outweighs the thermally polarised state that produces the positive background peak. Bubbling times between 5 and 70 s were examined that contrast with the 20 s needed to see SABRE with acetonitrile or indazole.

We have also probed the effect of adding CD_3_OH and H_2_O to these CD_3_OD solutions in order to increase the proportion of CD_3_OH in solution. These results are detailed in the Supporting Information and reveal complex behaviour. When H_2_O is added, the indazole, acetonitrile, methanol and HOD signal gains all first increase in size before falling as the level of doping is increased. The origin of this fall with added H_2_O is likely to result from the reduced solubility of H_2_ in water and the associated decrease in ^1^H relaxation times.^25^, ^30^ A related series of solutions were then examined in the presence of HCl and NaOH. No significant changes in the level of proton indazole or solvent signal enhancement were evident. This contrasts with the results of Moreno *et al*. who saw an increased level of polarisation transfer into the solvent molecules in acid solution at low field.^[10d]^ We note though that it takes 4.8 s to move the sample from the external MC into the high‐field magnet where our measurements are made, and hence, relaxation could account for this difference.

### Effect of changing the ligand scaffold to *d*_22_‐IMes or *d*_22_‐SIMes on the level of signal amplification by reversible exchange shown by indazole

Upon replacing the protio form of these NHC ligands with their deuterated variants *d*_22_‐IMes^7^, ^22^ and *d*_22_‐SIMes, the levels of indazole and acetonitrile proton signal enhancements under SABRE changed as detailed in Table 1. For *d*_22_‐IMes, the maximum indazole proton signal enhancement was now obtained at 90 G rather than 70 G, and after 35 s of contact with *p‐*H_2_, rather than the 20 s used earlier. Its final value is, however, reduced from 115‐fold to just 82‐fold, and hence, it can be concluded that in this case, ^2^H‐labelling of the catalyst has a negative effect on the level of indazole polarisation that is achieved when compared with the IMes system. In contrast, the acetonitrile proton signal enhancement level increased very dramatically from the original 145‐fold value to 572‐fold, with transfer now taking place at 90 G rather than the original 80 G field value (190‐fold per proton). Hence, polarisation transfer into acetonitrile has become more facile, and as a consequence, it now receives the largest SABRE benefit.

**Table 1:** Overall proton signal enhancement values (fold) returned for indazole and CH_3_CN as a function of catalyst at 9.4 T using the polarisation transfer field detailed in brackets

	Enhancement (PTF)	
Catalyst	Indazole	Acetonitrile
IMes	116 (70 G)	146 (80 G)
*d*_22_‐IMes	82 (90 G)	572 (90 G)
SIMes	294 (70 G)	437 (80 G)
*d*_22_‐SIMes	457 (70 G)	372 (80 G)

The associated reagent concentrations were 6.5 mM (iridium), 65 mM (indazole) and 19.5 mM (acetonitrile).

The corresponding change to *d*_22_‐SIMes, however, resulted in an indazole signal gain that averaged to ~91‐fold per proton, and a 372‐fold CH_3_CN signal gain at the same transfer field values as used earlier, which remained optimal. Deuterating the carbene ligand of **1a** therefore leads to a 41% fall in indazole polarisation, but for **1b**, it leads to a 60% increase. We note that increases in temperature result in further increases in these signal gains as the ligand exchange rates are slow relative to what might be expected to be their optimum values.^9^ In contrast, deuterating the IMes ligand associated with **2a** leads to a 300% gain in acetonitrile polarisation, whilst for **2b**, it leads to a 17% fall. This difference will be rationalised later.

### Effect of polarisation transfer field on the level of signal amplification by reversible exchange shown by indazole

**Figure 5:**
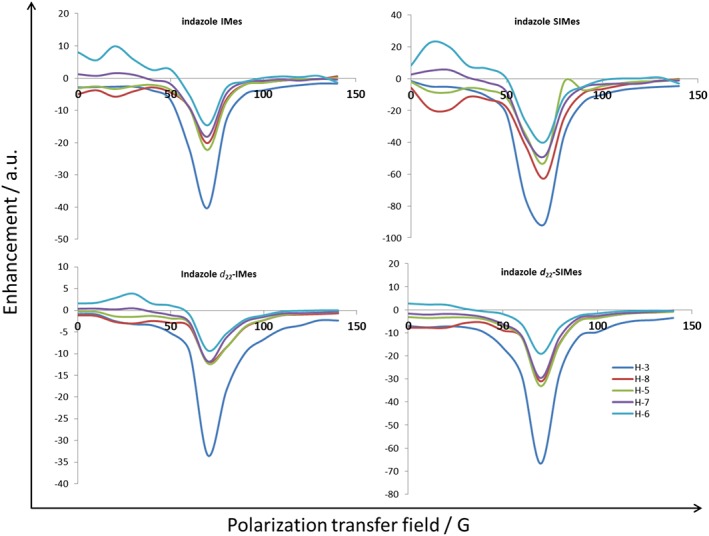
Polarisation transfer field plot showing how the indicated ^1^H‐NMR signal intensity gains seen for indazole change with catalyst ligand deuteration.

Figure 5 illustrates how the PTF affects the indazole proton enhancement level as a function of ligand scaffold deuteration. In the case of **3**, there is one unique hydride coupling into the bound indazole ligand that will lead to transfer to proton H‐3 of Scheme 1; the hydride couplings across the bridge into the second phenyl ring are expected to be too small to receive direct polarisation transfer via the hydride ligand. Protons H‐8, H‐5, H‐7 and H‐6 will therefore achieve their hyperpolarised states *via* relayed transfer through H‐3. This behaviour accounts for the fact that when the SABRE efficiency of **3** is examined, as a function of PTF, a signal maximum is evident for all of these protons (Fig. 5). The breadth of the peak seen in the PTF profile changes, narrowing with deuteration of the NHC, and hence, we conclude that the matching transfer condition narrows when the deuterated ligands are employed. These changes mean that there is a greater need to place the sample in an appropriate PTF when undertaking studies with deuterated ligands.

### Effect of relaxation within the signal amplification by reversible exchange catalyst on the level of indazole signal enhancement

An effect that needs to be considered when rationalising these results is that of relaxation within the catalyst, which is predicted to be reduced by deuteration. The importance of this stems from the fact that the bound and free forms of the ligands are in dynamic exchange, and hence, any increase in relaxation time of the associated protons in the catalyst should be seen directly in the response of the free substrate. For example, if the lifetime of the catalyst is too long, the associated signals will suffer from greater relaxation during the polarisation transfer step, and hence, increasing the catalysts' intrinsic proton relaxation times should be beneficial. In contrast, if ligand exchange is rapid, then the relaxation times of the free substrate will reflect more closely those of the bound form and that will act to limit the period over which hyperpolarised signals for the free substrate can be seen.

The relaxation times of the five protons of indazole and acetonitrile in a methanol‐*d*_4_ solution under 3 bar H_2_ without the catalyst at 298 K were therefore determined. They are 22.2 s for proton H‐3, 12.8 s for H‐8, 12.5 s for H‐5, 8.5 s for H‐7 and 9.3 s for H‐6 with the acetonitrile value being 14.4 s at 9.4 T. Upon adding the catalyst and H_2_ to this solution, and repeating the associated measurements at 298 K, the apparent relaxation time for proton H‐3 of free indazole, and that of the free acetonitrile resonance, was found to decrease to 12.3 and 8.7 s respectively, whilst those for the remaining four indazole sites fell by a smaller amount (Table 2). We confirm therefore that the presence of the catalyst reduces the measured relaxation times of the free substrate in solution and therefore acts to limit the period over which it can be viewed with a high‐sensitivity response.

**Table 2:** Experimental *T*_1_ values determined at 9.4 T in methanol‐*d*_4_ solution for the indicated proton nuclei of free and bound indazole at 263 and 298 K respectively

Site 263 K	IMes *T*_1_ (s)		*d*_22_‐IMes *T*_1_ (s)		SIMes *T*_1_ (s)		*d*_22_‐SIMes *T*_1_ (s)	
	Free	Coord	Free	Coord	Free	Coord	Free	Coord
H‐3	15 ± 4^a^	0.9	4.8	0.8	16.1	1.3	10.7	0.9
H‐8	7.0	^b^	3.5	1.4	8.4	2.7	5.8	2.2
H‐5	7.0	1.9	3.3	1.6	7.3	—	5.5	—
H‐7	4.1	2.2	2.6	—	4.6	1.7	3.6	1.1
H‐6	^b^	1.7	2.7	0.9	4.8	1.9	3.6	1.7
NCMe	8.2	—	4.8	—	11.5	1.3	8.2	—
Site 298 K	—							
H‐3	13.6	2.1	9.8	1.9	11.8	0.6	11.7	5.6
H‐8	10.3	5.8	7.4	3.7	10.8	—	8.9	4.2
H‐5	10.5	2.7	7.5	—	10.9	2.9	9.0	—
H‐7	6.7	4.0	5.5	2.3	7.0	1.7	6.2	3.75
H‐6	7.2	—	5.6	—	7.6	—	6.6	1.56
NCMe	8.7	—	8.9	—	12.4	—	10.2	—

The associated reagent concentrations were 6.5 mM (iridium), 65 mM (indazole) and 19.5 mM (acetonitrile), and the labelling follows that in Scheme 1.

a Peak behaviour anomalous due to ^2^H label incorporation.

Peak overlap prevents accurate assessment.

Cooling the sample to reduce the associated ligand exchange rates would be expected to suppress this relaxation‐based behaviour. We therefore undertook a control measurement on indazole at 263 K in methanol‐*d*_4_ at the same 50 mM concentration. The corresponding *T*_1_ values were now 17.4, 7.2, 7.2, 4.0 and 4.5 s respectively. Hence, as expected, lowering of the temperature reduces the *T*_1_ values of indazole.^31^ The corresponding *T*_1_ values were then determined for the protons of bound and free indazole in a mixture with **3** at 263 K. Now, as predicted, the corresponding bound proton values proved to be dramatically smaller than those of the free substrate, which, in turn, were remarkably similar to those determined without **3**. The size of the relaxation effects seen for **3b** proved to be even larger at 298 K in accordance with the higher ligand exchange rates exhibited by this complex, and we therefore conclude that relaxation of the hyperpolarisation prior to ligand dissociation is limiting. The corresponding relaxation data for the catalysts containing *d*_22_‐SIMes or *d*_22_‐IMes ligands were also determined and found to differ from those of their protio counterparts as shown in Table 2. We note that even though these parameters have been determined at high field, and the transfer process occurs at low field where the relaxation times will be different, a substantial effect has been seen. Given that the ligand exchange rates would not be predicted to change with remote ^2^H labelling, and the hydride‐proton couplings should also remain constant, this behaviour suggests that the ancillary ligands of the catalyst play a role through relaxation during the SABRE process.

### Polarisation transfer into the ^13^C response of indazole, acetonitrile and methanol

**Figure 6:**
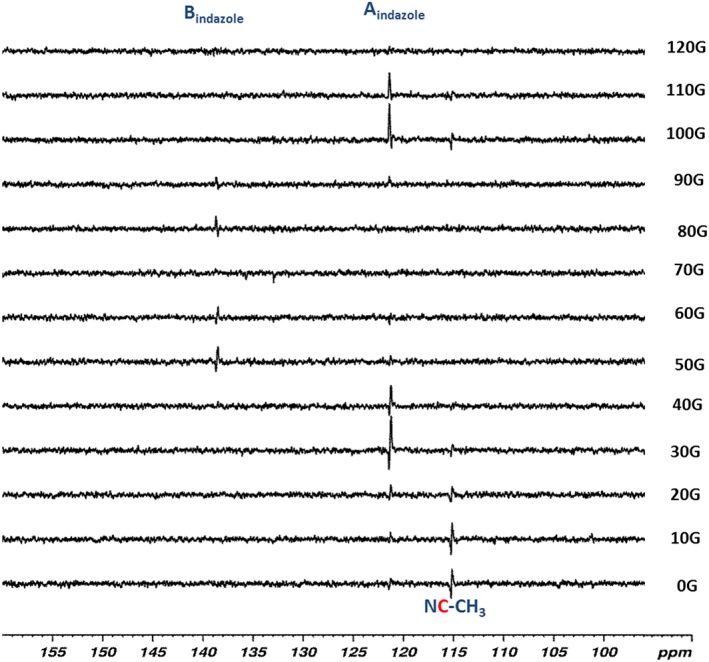
Polarisation transfer field effects seen on the intensity of the ^13^C NMR signals of indazole and NCCH_3_ that result from signal amplification by reversible exchange.

SABRE is also evident in the ^13^C resonances of indazole, acetonitrile and methanol as detailed in Fig. 6 for a 35‐s contact time with *p*‐H_2_ and the **1a**‐derived catalyst system. Polarisation transfer into the quaternary ^13^C signal for C_A_ of indazole reaches a maximum at 40 G (132‐fold) with transfer into quaternary C_B_ being observed between 50 (58‐fold) and 60 G, and 80 and 90 G, although there is a minimum at 70 G. The effect of the PTF on ^13^C transfer is therefore complex and reflects the number of different coupling pathways in operation. The size of the SABRE effect is PTF dependant with the ^13^C quaternary signal of free acetonitrile readily appearing at 116 ppm after transfer a ~0G in a μ‐metal shield (a ^15^N signal can also be seen, refer to Supporting Information). Upon increasing the transfer field beyond this value, the acetonitrile signal gain reduces, but at points beyond 100 G, it is again observed through an enhanced signal. We note that we do not observe polarisation transfer into the ^13^CH_3_ group of acetonitrile nor into the ^13^C atoms of methanol‐*d*_4_ under these conditions. This is consistent with earlier reports that detail how optimal ^13^C transfer proceeds via small ^3^ *J*_CH_ couplings rather than the larger ^2^ *J*_CH_ couplings.^22^

The levels of ^13^C signal gain achieved with **1b** proved smaller than those with **1a** as detailed in the Supporting Information, and the replacement of the SIMes or IMes ligands with *d*_22_‐SIMes and *d*_22_‐IMes respectively also led to worse SABRE performance with the maximum ^13^C signal enhancement seen for site A (Scheme 1) being 63‐fold in the latter case; adding acid into this system did not improved these signal enhancement levels.

### Reaction of IrCl(NHC)(COD) with imidazole, H_2_ and acetonitrile and the observation of signal amplification by reversible exchange

[IrCl(COD)(IMes)] (**1a**) and [IrCl(COD)(SIMes)] (**1b**) were then used to hyperpolarise imidazole (im). Similar 5 mM methanol‐*d*_4_ solutions of **1** or **2** were employed, with a 10‐fold excess of imidazole and a 3‐fold excess of acetonitrile. For **1a**, 97% of the products were now in the form [Ir(H)_2_(im)_3_(IMes)]Cl (**5a**) with the remaining 3% corresponding to [Ir(H)_2_(NCMe)(im)_2_(IMes)]Cl (**4a**) as detailed in Scheme 2. In the case of **1b**, the reaction proceeded cleanly to form **5b** as the sole product. Characterisation data for these products are detailed in the Supporting Information. When the SABRE effect was explored for imidazole, *p*‐H_2_ contact times of 60 s were used, rather than the 25–30‐s time for indazole, as this resulted in the detection of larger signal enhancements**.**

### Effect of polarisation transfer field on signal amplification by reversible exchange with imidazole

**Figure 7:**
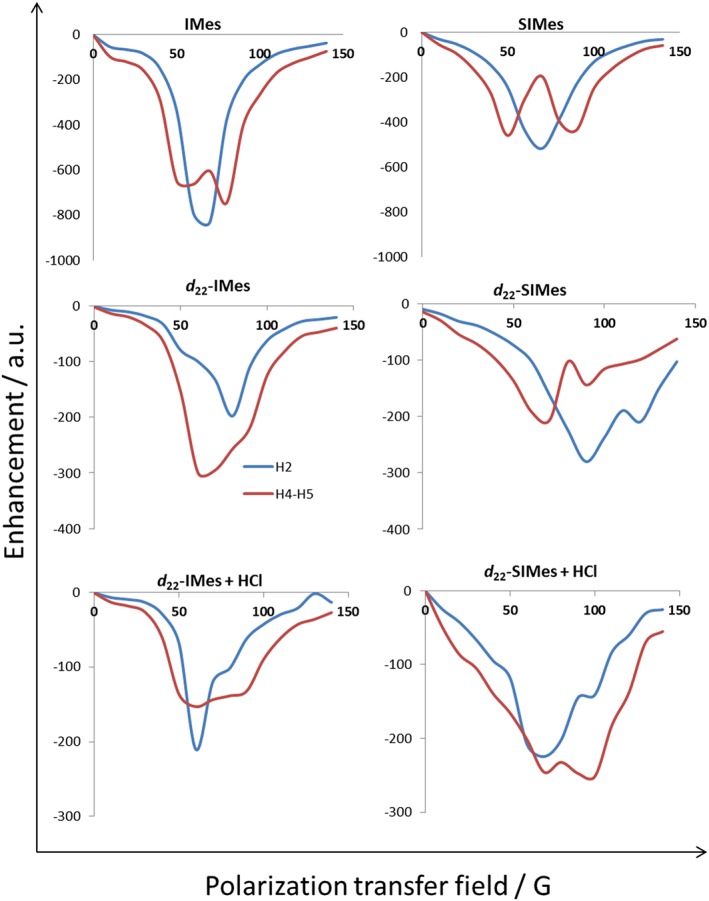
Polarisation transfer field plot showing how the indicated ^1^H‐NMR signal intensity gains seen for imidazole change with catalyst ligand deuteration and the introduction of acid.

**Scheme 3:**
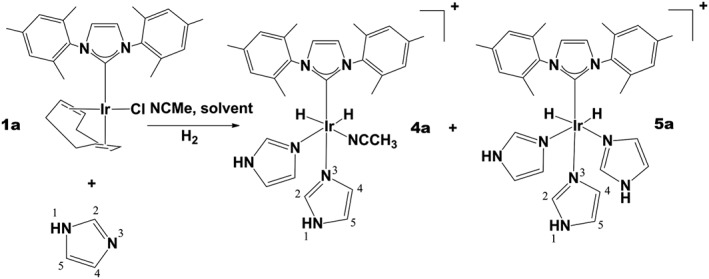
Formation of [Ir(H)_2_(im)_2_(IMes)(NCMe)]Cl (**4a**) and [Ir(H)_2_(im)_3_(IMes)]Cl (**5a**) via reaction of H_2_, acetonitrile and imidazole with **1a**, labels as used in the text.

Figure 7 illustrates how the PTF affects the imidazole proton signal enhancement level as a function of catalyst. In **5**, there are three unique spin–spin couplings between the hydride ligand (*trans*) and the imidazole protons in the SABRE transfer catalysts of Scheme 3 and six routes to relayed transfer between the three protons.^32^ These three protons exhibit chemical shifts of *δ*_H_ 6.82, 6.86 (H‐4 and H‐5) and 6.9 (H‐2) in **5a** and will all receive polarisation under SABRE with different efficiencies according to the PTF and couplings they experiences at the point of transfer. Consequently, upon the dissociation of hyperpolarised imidazole from **5**, all three of its proton signals should appear enhanced, but only two signals are actually seen, at *δ*_H_ 7.70 and 7.07, due to the fact the resonances for H‐4 and H‐5 have effectively identical chemical shifts. As expected, the SABRE results, with imidazole, therefore show a strong PTF dependence as detailed in Fig. 7. For H‐2, a signal peak is seen that reaches a maximum at a PTF value of 70 G, whilst the combination signal for H‐4 and H‐5 now shows a bimodal result with maxima at 50 and 90 G.

### Level of signal amplification by reversible exchange found for imidazole

In methanol‐*d*_4_, the **1a**‐derived catalyst system proved to deliver signal enhancements of 606‐fold and 830‐fold for these two signals respectively per proton, which equates to a total signal enhancement value of 2042‐fold within im. It would therefore appear that the NCHN motif, rather than the CHCH arrangement, receives the highest level of SABRE transfer. The overall signal gain clearly exceeds the level seen for indazole where the corresponding value was just 115‐fold, as detailed in Table 4. Furthermore, upon moving to **1b**, the overall signal enhancement falls to 1028‐fold that contrasts with the indazole return of 146‐fold, and the CHCH arrangement receives the most benefit. We can therefore conclude that imidazole is better suited to SABRE than indazole.

We also undertook a series of control measurements under analogous conditions without acetonitrile. The corresponding H‐2 signal of imidazole was now observed to yield a 551‐fold signal again under these conditions after transfer at 60 G, whilst the signals for H‐4/5 yielded a 410‐fold gain after transfer at 70 G per proton. Hence, imidazole polarises better in the presence of acetonitrile.

These results also proved to be pH sensitive, with the overall proton signal enhancement seen for imidazole dropping to 1475‐fold (H‐2, 571‐fold) with **1a** in the presence of HCl_(aq)_ as summarised in Table 3. Additionally, the acetonitrile signal gains, relative to those seen with indazole, proved to be much lower in accordance with the reduced concentrations of the associated catalyst **4**. The level of SABRE exhibited by the solvent CHD_2_OD is also worse than that seen with indazole in accordance with **4** that plays a role in this process, but can be improved 20‐fold by ^2^H labelling the IMes ligand.

**Table 3:** Indicated proton signal enhancement levels achieved at 298 K, with a PTF of 80 G, when measured at 9.4 T, by the action of the precatalysts **1a** and **1b** on imidazole, with and without HCl, where the concentrations are 6.5 mM iridium, 65 mM indazole and 19.5 mM acetonitrile

	^1^H signal enhancement (fold)			
Resonance (without HCl)	IMes	*d*_22_‐IMes	SIMes	*d*_22_‐SIMes
H‐2 H‐4 and H‐5	−830	−198	−517	−280
	−606	−300	−197	−206
CD_3_OH	1.5	10	1.4	1.2
CHD_2_OD	2.5	45	7.5	0.5
NCMe	−35	−30	−4	0
Resonance (with 5 mM HCl)	IMes	*d*_22_‐IMes	SIMes	*d*_22_‐SIMes
H‐2 H‐4 and H‐5	−571	−210	−647	−225
	−456	−153	−562	−251
CD_3_OH	−22	−13	−7	−31
CHD_2_OD	2	0.2	1.5	−0.5
NCMe	−16	−168	−12	11

### Imidazole ligand exchange rates in the active signal amplification by reversible exchange catalyst

The free imidazole build‐up rate in solution of **5a**, without HCl, proved to be 3.39 s^−1^ and its H_2_ loss rate 3.68 s^−1^. However, in the presence of 0.8 equivalent HCl, the imidazole build‐up rate in solution fell to 2.52 s^−1^, whilst the apparent H_2_ loss rate became 4.75 s^−1^. For **5b**, without HCl, the ligand build‐up rate was 1.04 s^−1^ and the H_2_ loss rate 1.75 s^−1^, but with HCl (0.8 equivalents), they became 1.11 and 1.85 s^−1^ respectively. These ligand build‐up rates are therefore faster than those seen for indazole and that must account for the improved SABRE performance seen with imidazole. However, it is interesting to see that the IMes system now exchanges more rapidly than the SIMes form which that suggests the buried volume^9^, ^33^ term associated with the NHC plays a greater role here in promoting ligand loss. Promotion of H_2_ loss in acid solution suggests that the complex is susceptible to protonation that would be detrimental to SABRE.^[10d]^

### Effect of changing the ligand scaffold to *d*_22_‐IMes or *d*_22_‐SIMes on the level of signal amplification by reversible exchange shown by imidazole

When the catalysts *d*_22_‐IMes‐**1a** and *d*_22_‐SIMes‐**1b** are deployed with imidazole, the signal intensities seen under SABRE in indazole and acetonitrile fall dramatically.

### Effect of relaxation within the signal amplification by reversible exchange catalyst on the level of imidazole signal enhancement

The relaxation times of the protons in imidazole were also studied in a similar way to those of indazole and are presented in Tables 4 and 5. The corresponding values for the free substrates in degassed methanol‐d_*4*_ solution are 51.1 s for H‐2 and 26.6 s for the H‐4 and H‐5 response of imidazole, whilst the value for CH_3_CN was 16.3 s at 298 K (the corresponding values at 263 K are 33.3, 13.5 and 11.4 s respectively). It is notable that the relaxation times for free imidazole are larger than those of indazole, with the N‐isolated proton H‐2 having a value of 51 s. At 298 K, this value falls by 67% to 16.9 s in the presence of **5a**, whilst the corresponding fall in indazole H‐2 *T*_1_ value was 39% under analogous conditions and is consistent with the higher ligand exchange rates for imidazole.

**Table 4:** Experimental *T*_1_ values, determined at 9.4 T in methanol‐*d*_4_ solution for the indicated proton nuclei of free and bound imidazole (**5b**) at 263 and 298 K respectively

Temperature		263 K		298 K		298 K (with HCl)	
Catalyst	Proton/*T*_1_ (s)	IMes	*d*_22_‐IMes	IMes	*d*_22_‐IMes	IMes	*d*_22_‐IMes
Free substrates	H‐2	33.7	25.5	16.9	22.9	15.8	21.8
	H‐4 and H‐5	14.1	12.0	12.2	15.1	11.9	14.8
	NCMe	8.2	9.6	8.3	9.5	7.9	10.3
Bound imidazole	H‐2_eq_	3.1	4.3	13.7	14.1	8.7	13.4
	H‐4_eq_	1.6	2.4	13.6	17.4	11.2	17.8
	H‐5_eq_	1.0	1.2	9.3	9.4	3.2	6.6
	H‐2_ax_	4.1	3.1	4.0	3.0	4.4	7.6
	H‐4_ax_	2.7	3.6	7.9	3.5	9.5	7.1
	H‐5_ax_	2.8	1.7	4.6	2.5	5.5	4.9
Temperature		263 K		298 K		298 K (with HCl)	
Catalyst	Proton/*T*_1_ (s)	SIMes	*d*_22_‐SIMes	SIMes	*d*_22_‐SIMes	SIMes	*d*_22_‐SIMes
Free substrates	H‐2	34.4	25.9	17.0	14.8	13.2	12.8
	H‐4 and H‐5	14.7	13.0	12.8	14.0	10.5	12.1
	NCMe	11.5	10.8	9.8	14.7	8.2	14.3
Bound imidazole (eq = equatorial) (ax = axial)	H‐2_eq_	3.5	3.7	13.0	11.0	13.1	9.9
	H‐4_eq_	1.8	1.4	11.1	10.0	11.0	10.2
	H‐5_eq_	1.9	1.9	9.7	8.8	10.6	10.5
	H‐2_ax_	—	3.0	—	8.2	—	—
	H‐4_ax_	2.8	3.6	7.9	6.0	17.9	7.5
	H‐5_ax_	—	2.6	4.2	3.8	13.8	4.8

The associated reagent concentrations were 6.5 mM (iridium), 65 mM (imidazole) and 19.5 mM (acetonitrile), and the labelling follows that in Scheme 1.

**Table 5:** ^13^C‐NMR signal enhancement levels seen for the indicated carbon signals of imidazole as a function of catalyst (optimum polarisation transfer field in brackets) at 9.4 T, with and without 5 mM HCl

Catalyst ^13^C signal	C_A_	C_B_	C_A_	C_B_
	Without HCl		With HCl	
IMes	61 (90 G)	9 (120 G)	59 (90 G)	40 (30 G)
*d*_22_‐IMes	243 (90 G)	—	144 (80 G)	149 (40 G)
SIMes	446 (100 G)	89 (100 G)	365 (90 G)	489 (110 G)
*d*_22_‐SIMes	274 (110 G)	—	221 (30 G)	322 (30 G)

reagent concentrations were 6.5 mM (iridium), 65 mM (imidazole) and 19.5 mM (acetonitrile).

For IMes, at 263 K, the relaxation times within **5a** of the three magnetisation receptors are 3.1 s (H‐2), 1.6 and 1.0 s (H‐4 and H‐5), whilst for **5b**, they are 3.5, 1.8 and 1.9 s respectively, and hence, all are larger than the 0.9‐s value determined for H‐3 in **3a**. This behaviour is therefore consistent with the improvement in SABRE efficiency that is seen on moving to imidazole. In the ^2^H‐labelled form of **5a**, the corresponding *T*_1_ values increase to 4.3, 2.4 and 1.2 s respectively, whilst in **5b**, they become 3.7, 1.4 and 1.9 s. Both of these sets of values at 9.4 T are therefore larger than those resulting from the corresponding ^1^H‐labelled catalysts. It is therefore clear that these high‐field values are not a good indicator of the role relaxation plays during SABRE transfer in low field.

### Polarisation transfer to ^13^C in imidazole

**Figure 8:**
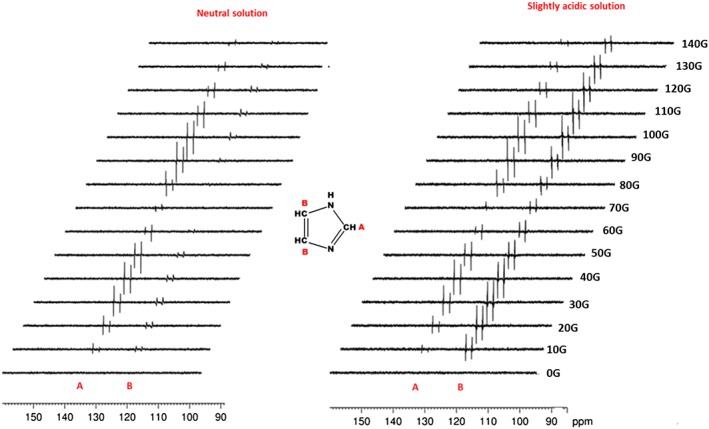
^13^C‐NMR signal amplification by reversible exchange intensities as a function of PTF before and after adding 0.8 equivalents of HCl_aq_ to 3 ml *d*_4_‐methanol solution of **1b**, with 7 equivalents of imidazole and 3 equivalents of NCMe at room temperature.

A series of hyperpolarised imidazole ^13^C‐NMR spectra were also recorded using a methanol‐*d*_4_ solution that initially contained a 7‐fold imidazole and 3‐fold acetonitrile excess relative to **1a**, with and without HCl. These NMR spectra are detailed in Fig. 8. The ^13^C signals of free imidazole also proved to exhibit far greater signal gains than those found for indazole. For the signal due to C_A_, a strong response was seen, whilst the averaged signal, observed for the two remaining CH sites, was weaker. The intensities exhibited by these signals are again field dependant with both showing maxima after transfer at ca. 40 and 90 G. In contrast, upon adding acid, the signals for C_B_ appear strongly (Fig. 8 right), presumably due to a sharpening of the response due to the increased rate of NH site interchange, although no effect is seen in the PTF plot. When **1b** is employed, the corresponding signal gains on C_B_ were around 10 times better than those seen with **1a** in neutral solution. When acid was added, the level of ^13^C response resulting from **1b** increased by a further 12‐fold. We can therefore conclude that whilst IMes is the better motif for ^1^H‐SABRE, ^13^C‐SABRE benefits from deploying SIMes. When the corresponding *d*_22_‐IMes and *d*_22_‐SIMes forms are examined, no real benefits are seen in neutral solution, but in slightly acidic solution, *d*_22_‐SIMes did yield a further doubling in ^13^C polarisation level. It is therefore clear that the optimal catalyst for SABRE depends on the NMR active nucleus that is targeted. We note that ^15^N signals can also be seen after transfer in the μ‐metal shield as described in the Supporting Information.

## Conclusions

In conclusion, we have described a series of studies on the SABRE hyperpolarisation of the substrates indazole and imidazole, in the presence and absence of the co‐ligand acetonitrile, using the precatalysts [IrCl(COD)(IMes)] and [IrCl(COD)(SIMes)]. These studies reveal that SABRE delivers a 830‐fold signal enhancement for the H‐3 resonance of imidazole and a 178‐fold improvement for the H‐2 resonance of indazole at 9.4 T in the presence of acetonitrile. These signal gains compare with values of 515 and 334‐fold that were observed here in the absence of acetonitrile and indicate that in the case of imidazole, the presence of a co‐ligand is beneficial. The co‐ligand acetonitrile was observed to exhibit a maximum 572‐fold signal gain (190‐fold per proton) during this study that reveals how this value is controlled by the identity of the substrate. If high acetonitrile polarisation is targeted, working in the presence of ^2^H‐labelled indazole is therefore likely to reflect a good solution.

These results have been rationalised by studies on the catalyst, which in this case revealed two complexes result, [Ir(H)_2_(sub)_2_(NCMe)(NHC)]Cl and [Ir(H)_2_(sub)_3_(NHC)]Cl. When the NHC is IMes or SIMes, the *tris*‐substituted complex is favoured over the *bis*‐substituted complex. Furthermore, both of these complexes undergo the necessary substrate and H_2_ loss processes that allow them to drive the hyperpolarisation of these agents. The H_2_ loss rates for the complexes with indazole were 0.65 s^−1^ for IMes and 0.08 s^−1^ for SIMes, whilst for imidazole, they were 3.68 and 1.75 s^−1^. Given that *p*‐H_2_ is the source of hyperpolarisation, these values suggest that indazole should show weaker polarisation than imidazole, and that the IMes form of the catalyst is superior. However, substrate loss rates also play a role in controlling the level of SABRE. In the case of **2a**, this is reflected in the bound acetonitrile and indazole ligands, whilst in **3a**, iwhilst in **3a**, it is just indazole ligand loss that is important. The effective ligand build‐up rates in solution for indazole were 0.24 s^−1^ in **3a** and compare with that for imidazole of 3.39 s^−1^ in **5a**. Hence, the higher substrate loss rate matches with the observation of better SABRE performance in the hyperpolarisation of imidazole through **5a**. However, all three of these free ligand build‐up rates are substantially smaller than that exhibited for pyridine by [Ir(H)_2_(IMes)(pyridine)_3_]Cl, which is 22 s^−1^. The hyperpolarisation of indazole and imidazole reported here is therefore less efficient than that achieved for pyridine.^[6b]^

One further impact of these ligand exchange values is reflected in the optimum *p*‐H_2_ contact time, which is referred to here as the bubbling time when the automated polariser is used. Whilst polarisation transfer proceeds under J‐coupling, the catalyst gains its polarisation for transfer through the addition of fresh *p*‐H_2_. Hence, if the complex lifetime is short, *p*‐H_2_ addition and transfer must proceed on a relatively rapid timescale, and a short bubbling time can be employed. If the catalyst lifetime is longer, *p*‐H_2_ addition and transfer will proceed on a slower timescale, and a longer bubbling time can be employed to reach the point where relaxation acts to limit the ultimate level of polarisation that is exhibited by the free substrate. The recent paper by Barskiy et al. develops these points.^[11d]^ It is for this reason that we used a 20‐s bubbling time when detecting a ^1^H response for indazole, extending to 35 s when looking at the corresponding ^13^C signals, and 60 s for imidazole. Furthermore, upon changing the NHC to SIMes, the corresponding indazole build‐up rates increase to 0.64 s^−1^ for **3b** but fall to 1.04 s^−1^ for **5b**. This suggests that for indazole, SIMes works better due to higher exchange, but for imidazole, the reverse is found experimentally, and hence, the H_2_ exchange rate must be more critical.

We also utilised a series of ^2^H‐labelled forms of these catalysts with a view to improve on the level of SABRE by harnessing potentially longer catalyst relaxation times. An earlier report by Fekete et al.^7^ demonstrated how ^2^H labelling of the catalyst led to a dramatic increase in the level of SABRE. Barskiy et al. have built on these, and other results, to produce a simple analytical model to describe this behaviour that suggests that relaxation and not ligand dissociation is critical to achieving optimal enhancement.^[11d]^ The dominant mechanisms for relaxation within these small‐molecule system are predicted to be dipole–dipole based, with scalar relaxation through coupling to a second quadrupolar nucleus playing a role.^31^, ^34^ The SIMes system proved optimal for indazole, and the 90‐fold signal gain per proton with *d*_22_‐SIMes exceeded the performance of the ^1^H‐labelled form by 150%. In contrast, the better performing ^1^H form of IMes yielded a 680‐fold signal gain per proton in imidazole, but upon changing to its *d*_22_‐IMes counterpart, SABRE efficiency fell by 60%. Hence ^2^H labelling does not automatically lead to improved SABRE.

SABRE derived signals gains were also revealed in the ^13^C and ^15^N responses of these agents, which in the case of imidazole were improved by the addition of trace amounts of HCl to promote proton transfer, resulting in a sharp coalesced response for its two nominally inequivalent CH‐CH centres (Supporting Information).

Results were presented here that detail the relaxation effects of the active form of the catalyst. All of these catalysts proved to contain bound substrate molecules whose protons had relaxation times that were between 25 and 5 times smaller than those of the free material. For imidazole, the longer proton relaxation times that are exhibited by the free material were found to translate into longer relaxation times in the corresponding protons on the catalyst at field. If this situation is mimicked at low field where SABRE transfer takes place, this will act to minimise the loss of *p*‐H_2_‐derived hyperpolarisation during polarisation transfer. The use of ^2^H‐labelled NHCs in the form of *d*_22_‐IMes‐**1a** and *d*_22_‐SIMes‐**1b**, however, resulted in a slight reduction in these relaxation times. It is noteworthy, however, that the level of acetonitrile hyperpolarisation was dramatically improved by using *d*_22_‐IMes rather than its ^1^H‐containing version by over 390%. These results serve therefore to illustrate the complexity of this process and suggest that a series of rigorous experimental studies are needed to produce a truly optimised catalyst for a specific substrate.

## Supporting information

Data S1. Supporting info item
