# Supplementary material for: Harnessing polarisation transfer to indazole and imidazole through signal amplification by reversible exchange to improve their NMR detectability
Source: Magn Reson Chem. 2017 Jun 20;55(10):944–57. doi: 10.1002/mrc.4607 (PMC5599957; doi:10.1002/mrc.4607)
Supplement: Supplementary file 1 — Data S1. Supporting info item [file MRC-55-944-s001.docx]

**Supporting information**

**Harnessing Polarization Transfer to Indazole and Imidazole Through Signal Amplification by Reversible Exchange to Improve their NMR Detectability**

Marianna Fekete, Peter J. Rayner, Gary. G. R. Green and Simon B. Duckett*

Centre for Hyperpolarization in Magnetic Resonance, Department of Chemistry, University of York, York, YO10 5NY, UK, email simon.duckett@york.ac.uk

Contents

[1. General 4](#_Toc480896193)S

[2. Synthetic procedures and Characterisation data for [IrCl(COD)(*d*_22_-SIMes)] 5](#_Toc480896194)S

[3. Characterisation data for 2a and 3a 7](#_Toc480896203)S

[4. Characterisation data for 2b and 3b 8](#_Toc480896204)S

[5. Polarization transfer field profile for the SABRE enhancement of the ^1^H NMR signals of indazole achieved by the precataysts [IrCl(IMes)(COD)] and [IrCl(*d*_22_-IMes)(COD)]. 9](#_Toc480896205)S

[Figure S1. Polarization transfer field profile for the SABRE enhancement values determined for the indicated ^1^H NMR signals of indazole achieved using the precataysts: a) IrCl(IMes)(COD) and b) IrCl(*d*_22_-IMes)(COD). 9](#_Toc480896206)S

[6. Polarization transfer field profile for the SABRE enhancement values determined for the ^1^H NMR signals of indazole by precatayst [ IrCl(SIMes)(COD)]. 10](#_Toc480896207)S

[Figure S2. Polarization transfer field profile for the SABRE enhancement values determined for the ^1^H NMR signals of indazole achieved using the precataysts: a) [IrCl(SIMes)(COD)] and b) [IrCl(*d*_22_-SIMes)(COD)]. 10](#_Toc480896208)S

[7. Polarization transfer field profiles for the acetonitrile ^1^H NMR signal gains seen in solution with indazole using [IrCl(IMes)(COD)], [IrCl(d_22_-IMes)(COD)], [IrCl(SIMes)(COD)] and [IrCl(d_22_-SIMes)(COD)]. 11](#_Toc480896209)S

[Figure S3: Polarisation transfer field plots showing how the ^1^H NMR signal intensity gains seen for NCMe change with catalyst, the catalysts are labelled in the figure. 11](#_Toc480896210)S

[8. Signal enhancement values achieved for the indicated substrate and solvent protons after transfer at 70 G as a function of H_2_O or CD_3_OH loading. 12](#_Toc480896211)S

[Figure S4. Spectroscopic examination of ^1^H NMR signals of residual CD_3_OH, CHD_2_OD and H_2_O seen under SABRE. 13](#_Toc480896212)S

[9. Normalised ^1^H NMR signal enhancement levels, measured in the presence of water (M) and additional CD_3_OH after transfer at 70 G. 14](#_Toc480896213)S

[10. SABRE derived ^13^C NMR signal enhancements seen for indazole as a function of the polarization transfer field. 15](#_Toc480896214)S

[11. ^15^N NMR signal enhancement seen for NCMe. 16](#_Toc480896215)S

[12. Characterisation data for 5a. 17](#_Toc480896216)S

[13. Characterisation data for 5b. 18](#_Toc480896217)S

[14. Relaxed and polarized ^1^H-NMR spectra in methanol-*d*_4_ of imidazole. 19](#_Toc480896218)S

[15. Polarization transfer field profile for the SABRE enhancement seen for the ^1^H NMR signals of imidazole as achieved using the precataysts [IrCl(IMes)(COD)] and [IrCl(*d*_22_-IMes)(COD)]. 20](#_Toc480896219)S

[Figure S8. Polarization transfer field profile for the SABRE enhancement of the ^1^H NMR signals of imidazole achieved using the precatayst: a) [IrCl(IMes)(COD)] and b) [IrCl(*d*_22_-IMes)(COD)]. 20](#_Toc480896220)S

[16. Polarization transfer field profile for the SABRE enhancement seen for the ^1^H NMR signals of imidazole achieved using the precataysts [IrCl(SIMes)(COD)] and [IrCl(*d*_22_-SIMes)(COD)]. 21](#_Toc480896221)S

[Figure S9. Polarization transfer field profile for the SABRE enhancement values seen for the ^1^H NMR signals of imidazole achieved using the precataysts: a) [IrCl(SIMes)(COD)] and b) [IrCl(*d*_22_-SIMes)(COD)]. 21](#_Toc480896222)S

[17. Polarization transfer field profile for the SABRE enhancement values determined for the ^1^H NMR signals of imidazole using the precataysts [IrCl(*d*_22_-IMes)(COD)] and [IrCl(*d*_22_-SIMes)(COD)] under acidic conditions. 22](#_Toc480896223)S

[Figure S10. Polarization transfer field profile for the SABRE enhancement values seen for the ^1^H NMR signals of imidazole achieved using the precataysts: a) [IrCl(*d*_22_-IMes)(COD)] and b) [IrCl(*d*_22_-SIMes)(COD)]. 22](#_Toc480896224)S

[18. Polarization transfer field profile for the acetonitrile ^1^H NMR signal response in solution with imidazole and [IrCl(IMes)(COD)], [IrCl(*d*_22_-IMes)(COD)], [IrCl(SIMes)(COD)] or [IrCl(*d*_22_-SIMes)(COD)]. 23](#_Toc480896225)

[19. SABRE derived ^13^C NMR signal enhancement data for imidazole. 24](#_Toc480896226)S

[20. ^15^N NMR signal enhancement on imidazole 25](#_Toc480896227)S

[21. References 26](#_Toc480896228)S

# General

The following compounds were synthesised according to literature procedures; [IrCl(COD)(IMes)] (**1**)^[^[^1^](#_ENREF_1)^]^ and [IrCl(COD)(*d*_22_-IMes)].^[^[^2^](#_ENREF_2)^]^ Indazole and imidazole (L^1^) and methanol-*d*_4_ were purchased from Sigma-Aldrich and used as received.

NMR samples were made up that contained **1a**, and **L^1^**, in a ratio of 1 : 8 in deuterated methanol (total volume 0.6 ml (Young’s capped NMR tube experiment) or 3 ml (NMR flow system experiment). Young’s capped NMR tubes were degassed three times on a Schlenk line whilst immersing the solution in a dry CO_2_/acetone slush bath. *Para*hydrogen was introduced into the NMR tube using home-built equipment; the *para*hydrogen was formed by cooling the dihydrogen gas to 36 K over a bed of Fe_2_O_3_.^[^[^3^](#_ENREF_3)^]^ For flow measurements, *para*hydrogen was supplied by a Bruker *para*hydrogen generator.

For the hyperpolarisation experiments, *para*hydrogen was either bubbled through the solution for a specified time (flow measurements)^[^[^4^](#_ENREF_4)^]^ or the sample was shaken in the fringe field of the magnet. The magnetic field in which *para*hydrogen was introduced into the sample was measured using a Hall probe.

NMR data was collected on a Bruker Advance III NMR spectrometer at 9.4 T.

Signal enhancement values were calculated by reference to the integrals of the corresponding thermally polarised peaks for the protons of **L^1^**, relative to those after hyperpolarisation transfer, at the magnetic field indicated. They are un-corrected for relaxation losses due to the transfer time (2.9 s for flow measurements and ≈ 4 s for Young’s capped NMR tube experiments) to the magnet.

# Synthetic procedures and Characterisation data for [IrCl(COD)(*d*_22_-SIMes)]

***d*_11_-2,4,6-trimethylaniline**

Acetic acid (1.80 mL) was added, dropwise, to a stirred solution of d_11_-nitromesitylene (783 mg, 4.45 mmol, 1.0 eq.) and zinc powder (1.47 g, 22.25 mmol, 5.0 eq.) in EtOH (15 mL) at 0 °C. The resulting solution was warmed to rt and stirred at rt for 5 h. Then, 1 M NaOH(aq) (10 mL) was added and the mixture was extracted with hexane (3 x 15 mL). The combined organic layers were dried (MgSO_4_) and concentrated under reduced pressure to give *d*_11_-2,4,6-trimethylaniline (583 mg, 90 %) as an orange oil, **^1^H NMR** (400 MHz, CDCl_3_) *δ* 3.37 (br s, 2H, NH_2_); **^13^C NMR** (100.6 MHz, CDCl_3_) *δ* 140.0 (CNH_2_), 128.7 (t, *J* = 22 Hz, CD), 126.9 (s), 121.7 (s), 19.4 (sept., *J* = 20 Hz, CD_3_), 16.7 (sept, *J* = 20 Hz, CD_3_); **MS** (ESI) m/z 147 [(M + H)^+^, 100]; **HRMS** m/z [M + H]^+^ calcd for C_9_H_3_D_11_N 147.1817, found 147.1819 (+0.3 ppm error). These spectroscopic values are consistent with those reported in the literature.^[^[^5^](#_ENREF_5)^]^

***d*_22_-2,4,6-Trimethyl-*N* -[2-[(2,4,6-trimethylphenyl)imino]ethylidene]aniline**

Glyoxal (160 µL of a 40 w/w% in H_2_O, 1.37 mmol, 1.0 eq.) and formic acid (2 drops) were added sequentially to a stirred solution of d_11_-mesitylaniline (400 mg, 2.74 mmol, 2.0 eq.) in MeOH at rt. The resulting solution was stirred at rt for 16 h during which time a yellow precipitate formed. The precipitate was filtered, washed with MeOH and dried under vacuum to give the ethylenediimine (293 mg, 68%) as a yellow crystalline solid, **^1^H NMR** (400 MHz, CDCl_3_) *δ* 8.12 (s, 2H); **^13^C NMR** (100.6 MHz, CDCl_3_) *δ* 163.4 (s), 147.5 (s), 134.0 (s), 128.7 (t, *J* = 22 Hz, CD), 126.4 (s), 19.9 (sept., *J* = 20 Hz, CD_3_), 17.3 (sept, *J* = 20 Hz, CD_3_); **MS** (ESI) m/z 337 [(M + Na)^+^, 30], 315 [(M + H)^+^, 100]; **HRMS** m/z [M + H]^+^ calcd for C_20_H_3_D_22_N_2_ 315.3393, found 315.3378 (+4.7 ppm error).

***d*_22_-1,3-Bis-(2,4,6-trimethylphenyl)imidazolinium chloride (*d*_22_-SIMes.HCl)**

NaBH_4_ (265 mg, 7.0 mmol, 4.1 eq.) was added to a stirred solution of the diimine (500 mg, 1.70 mmol, 1.0 eq.) in THF (10 mL) at 0 °C. Concentrated HCl (281 µL) was added, over the next 30 min, and the resulting solution was stirred at 0 °C for 20 min. 3 M HCl_(aq)_ (10 mL) was then added before warming to rt for 1 h. The precipitate was filtered and washed with water (15 mL) and 5% acetone/ether (15 mL) and dried under reduced pressure to give the intermediate diamine product as the dihydrochloride salt (659 mg) which required no further purification. Triethylorthoformate (1.78 mL, 10.71 mmol, 7.0 eq.) and formic acid (2 drops) were added sequentially to the diamine intermediate and the solution was heated to 120 °C for 5 h. The resulting solution was cooled to rt and hexane (20 ml) added. The resulting suspension was stirred at rt for 1 h. The precipitate was collected by filtration and washed with hexane (20 mL) and dried under reduced pressure to give *d*_22_-SIMes.HCl (281 mg, 45%) as an off white solid, **^1^H NMR** (400 MHz, CDCl_3_) *δ* 9.08 (br s, 1H), 4.48 (s, 4H); **^13^C NMR** (100.6 MHz, CDCl_3_) *δ* 159.8 (s), 139.4 (s), 134.8 (s), 129.8 (s), 129.4 (t, *J* = 24 Hz, CD), 51.5 (s), 20.3 (sept., *J* = 20 Hz, CD_3_), 17.3 (sept., *J* = 20 Hz, CD_3_); **MS** (ESI) m/z 329 [(M – Cl)^+^, 100]; **HRMS** m/z calcd for C_21_H_5_D_22_N_2_ (M – Cl)^+^ 329.3555, found 329.35551 (−2.7 ppm error).

**[IrCl(COD)(*d*_22_-SIMes)]**

KO^t^Bu (33 mg, 0.29 mmol, 2.4 eq.) was added to a stirred solution of *d*_22_-SIMes.HCl (100 mg, 0.27 mmol, 2.2 eq.) in THF at rt under N_2_. The resulting suspension was stirred at rt for 30 min. Then, a solution of [Ir(COD)Cl]_2_ (81 mg, 0.12 mmol, 1.0 eq.) was added and the resulting solution was stirred at rt for 2 h. The solvent was removed under reduced pressure to give the crude product. Purification by flash column chromatography on silica with CH_2_Cl_2_ as eluent gave [IrCl(COD)(*d*_22_-SIMes)] (100 mg, 63%) as a yellow crystalline solid, R_f_ (CH_2_Cl_2_) 0.2; **^1^H NMR** (400 MHz, CDCl_3_) *δ* 4.16-4.09 (m, 2H), 3.89 (s, 4H), 3.14-3.08 (m, 2H), 1.70-1.59 (m, 4H), 1.37-1.25 (m, 4H); **^13^C NMR** (100.6 MHz, CDCl_3_) *δ* 207.4 (s), 137.9 (s), 137.5 (s), 136.3 (s), 135.0 (s), 129.5 (t, *J* = 24 Hz, CD), 128.1 (t, *J* = 24 Hz, CD), 82.7 (s), 51.5 (s), 51.1 (s), 33.4 (s), 28.7 (s), 20.2 (sept., *J* = 20 Hz, CD_3_), 19.0 (sept., *J* = 19 Hz, CD_3_), 17.6 (sept., *J* = 20 Hz, CD_3_); **MS** (ESI) m/z 629 [(M(^193^Ir) – Cl)^+^, 100], 627 [(M(^191^Ir) – Cl)^+^, 60]; **HRMS** m/z calcd for C_29_H_16_D_22_^193^IrN_2_ (M – Cl)^+^ 629.4045, found 629.4041 (−3.5 ppm error).

# Characterisation data for 2a and 3a

**[Ir(H)_2_(NCCH_3_)(indazole)_2_(IMes)]Cl (2a)**: ^1^H NMR (500 MHz, MeOD, 298 K): δ -21.22 (1H, hydride *trans* to indazole), -20.80 (1H, hydride trans to NCCH_3_), 2.23 (3H, NCC*H*_3_), 2.25 (6H, C*H*_3_, IMes), 2.30 (6H, C*H*_3_, IMes), 2.34 (6H, C*H*_3_, IMes), 6.38 (4H, -C*H*=C*H*-, IMes), 7.05 (2H, -C*H*=C*H*-, IMes), 6.66 , 6.75, 7.14 (1H, indazole ax.), 7.27, 7.35, 7.85, 8.60 (1H, indazole eq.).

**[Ir(H)_2_(indazole)_3_(IMes)]Cl (3a)**: ^1^H NMR (500 MHz, MeOD, 298 K): δ -21.14 (2H, hydride), 2.00 (6H, C*H*_3_, IMes), 2.06 (12H, C*H*_3_, IMes), 6.38 (4H, -C*H*=C*H*-, IMes) ,7.04 (2H, -C*H*=C*H*-, IMes), 6.66, 6.88, 7.00, 7.40, 7.92 (1H, indazole in axial position), 7.16, 7.33, 7.50, 7.78, 8.53 (1H, indazole).

^13^C NMR (500 MHz, MeOD, 295 K): δ 17.2 (*C*H_3_, IMes), 19.8 (*C*H_3_, IMes), 108.8, 109.7 (*C*H, indazole in *ax*), 119.8 (CH, IMes), 120.9 (*C*H, indazole *ax*), 121.0 (CH, indazole in *eq*), 122.2, 125.1 (NCH, IMes), 122.5 (NCH, indazole in *ax*), 122.8 (NCH, indazole in *eq*), 126.3 (CH, indazole in *eq*), 126.4 (CH, indazole in *ax*), 134.4 (-C=, indazole in *ax*), 135.5 (C(CH_3_), IMes), 135.7 (-C=, indazole in *eq*), 137.9 (C(CH_3_), IMes), 138.5 (-C=, IMes), 148.9 (-C=, indazole in *ax*), 149.5 (-C=, indazole in *eq*), 161.8 (N*C*N, IMes).

# Characterisation data for 2b and 3b

**[Ir(H)_2_(NCCH_3_)(indazole)_2_(SIMes)]Cl (2b)**: ^1^H NMR (500 MHz, MeOD, 298 K): δ -21.29 (1H, hydride *trans* to indazole), -20.97 (1H, hydride trans to NCCH_3_), 2.09 (6H, C*H*_3_, SIMes), 2.21 (6H, C*H*_3_, SIMes), 2.375 (3H, NCCH_3_), 2.46 (6H, C*H*_3_, IMes), 3.78, 3.87 (4H, C*H*_2_, SIMes), 6.99 (4H, -C*H*=C*H*-, SIMes), 7.12, 7.36, 7.58, 7.79, 8.10 (1H, indazole in axial position), 5.63, 6.76, 7.19, 7.62, 8.28 (1H, indazole in equatorial position), 5.63, 6.65, 7.46, 7.93, 8.21 (1H, indazole in axial position).

**[Ir(H)_2_(indazole)_3_(SIMes)]Cl (3b)**: ^1^H NMR (500 MHz, MeOD, 298 K): δ -21.24 (2H, hydride), 1.98 (6H, C*H*_3_, SIMes), 2.26 (12H, C*H*_3_, SIMes), 3.81 (4H, -C*H*_2_-C*H*_2_-, SIMes), 6.38 (4H, -C*H*=C*H*-, SIMes), 6.70 (2H, -C*H*=C*H*-, SIMes), 7.12, 7.36, 7.58, 7.79, 8.10 (1H, indazole in axial position), 7.19, 7.33, 7.50, 7.84, 8.53 (1H, indazole in equatorial position). ^13^C NMR (500 MHz, 295 K): δ 17.49 (*C*H_3_, IMes), 19.81 (*C*H_3_, IMes), 50.03 (CH_2_, SiMes), 108.8, 109.7 (*C*H, indazole in *ax*), 119.84 (CH, IMes), 120.96 (*C*H, indazole *ax*), 121.04 (CH, indazole in *eq*), 122.42 (NCH, indazole in *ax*), 122.51 (NCH, indazole in *eq*), 126.32 (CH, indazole in *eq*), 126.37 (CH, indazole in *ax*), 134.45 (-C=, indazole in *ax*), 135.46 (C(CH_3_), SIMes), 135.75 (-C=, indazole in *eq*), 136.67 (C(CH_3_), SIMes), 137.80 (-C=, SIMes), 140.07 (-C=, indazole in *ax*), 140.89 (-C=, indazole in *eq*), 178.46 (N*C*N, SIMes).

# Polarization transfer field profile for the SABRE enhancement of the ^1^H NMR signals of indazole achieved by the precataysts [IrCl(IMes)(COD)] and [IrCl(*d*_22_-IMes)(COD)].

Sample parameters used to collect the data shown in Figure S1: 6.5 mM [Ir], 10 fold excess of indazole, 3 fold excess of NCMe based on iridium. Labelling according to the inset structure of indazole. Individual signal gains illustrated.

a)

b)

# Figure S1. Polarization transfer field profile for the SABRE enhancement values determined for the indicated ^1^H NMR signals of indazole achieved using the precataysts: a) IrCl(IMes)(COD) and b) IrCl(*d*_22_-IMes)(COD).

# Polarization transfer field profile for the SABRE enhancement values determined for the ^1^H NMR signals of indazole by precatayst [ IrCl(SIMes)(COD)].

Sample parameters used to collect the data of Figure S2: 6.5 mM [Ir], 10 fold indazole excess and 3 fold NCMe excess relative to iridium. Labelling according to the inset structure for indazole. Individual signal gains illustrated.

a)

b)

# Figure S2. Polarization transfer field profile for the SABRE enhancement values determined for the ^1^H NMR signals of indazole achieved using the precataysts: a) [IrCl(SIMes)(COD)] and b) [IrCl(*d*_22_-SIMes)(COD)].

# Polarization transfer field profiles for the acetonitrile ^1^H NMR signal gains seen in solution with indazole using [IrCl(IMes)(COD)], [IrCl(d_22_-IMes)(COD)], [IrCl(SIMes)(COD)] and [IrCl(d_22_-SIMes)(COD)].

Polarisation transfer field plots showing how the ^1^H NMR signal intensity gains seen for NCMe change with catalyst for samples based on 6.5 mM [Ir] and 10 equivalents of indazole and 3 equivalents of NCMe.


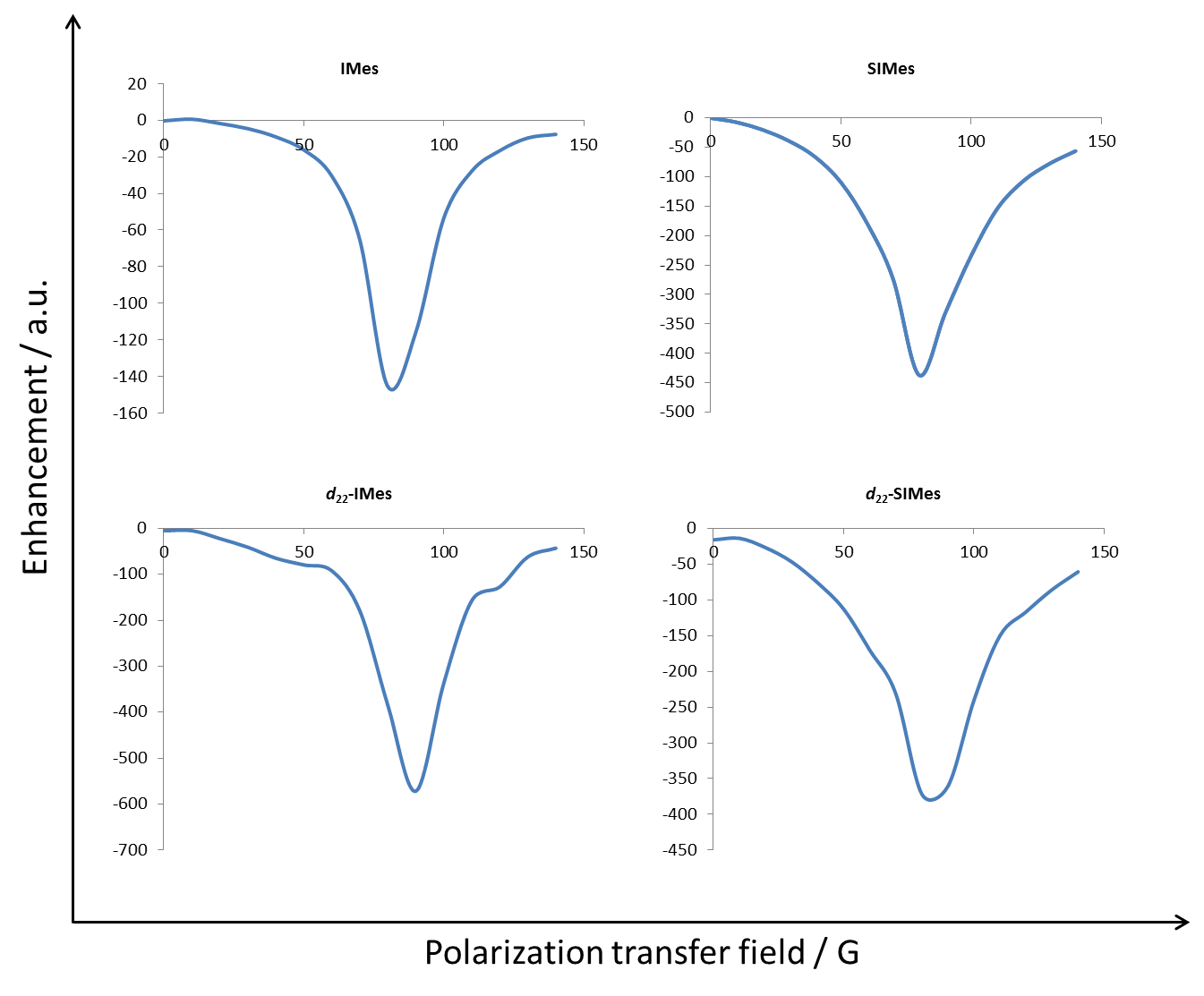


# Figure S3: Polarisation transfer field plots showing how the ^1^H NMR signal intensity gains seen for NCMe change with catalyst, the catalysts are labelled in the figure.

# Signal enhancement values achieved for the indicated substrate and solvent protons after transfer at 70 G as a function of H_2_O or CD_3_OH loading.

**Table S1** Normalised ^1^H NMR signal enhancement levels (per proton) measured in the presence of water (M) or additional CD_3_OH after transfer at 70 G.

| **Water**  **/M** | **^1^H NMR signal enhancement / a.u.** | | | |
| --- | --- | --- | --- | --- |
|  | **Indazole** | **CD_3_OH** | **NCMe** | **H_2_O** |
| 0  0.093 | 36.4 | 0.68 | 36.9 | 0 |
|  | 44.8 | 0.12 | 45.1 | 0.06 |
| 0.180  0.360 | 25.5 | 0.08 | 40.1 | 0.01 |
|  | 30.1 | 0.14 | 41.9 | 0.06 |
| 0.648  0.940 | 23.9 | 0.25 | 34.7 | 0.68 |
|  | 19.4 | 0.31 | 28.5 | 0.17 |
| 1.220  1.850 | 15.2 | 0.40 | 22.9 | 0.09 |
|  | 11.5 | 0.47 | 18.5 | 0.32 |
| **CD_3_OH**  **/M** | **Indazole** | **CD_3_OH** | **NCMe** |  |
| 0  0.093 | 36.9 | 0.72 | 39.5 |  |
|  | 81.0 | 0.72 | 50.8 |  |
| 0.180  0.360 | 48.4 | 0.20 | 38.0 |  |
|  | 46.4 | 0.18 | 37.6 |  |
| 0.648  0.940 | 13.3 | 0.73 | 2.8 |  |
|  | 11.1 | 0.53 | 1.9 |  |
| 1.220  1.850 | 10.3 | 0.44 | 2.1 |  |
|  | 6.82 | 0.38 | 2.1 |  |

Table S1 presents the normalised ^1^H NMR signal enhancement levels (per proton) measured in the presence of water (M) or additional CD_3_OH after transfer at 70 G. The initial solution contained 6.5mM of **1a** and 7-equivalents of indazole and 3 equivalents of NCMe relative to iridium. A 20 s *p*-H_2_ bubbling time was used.


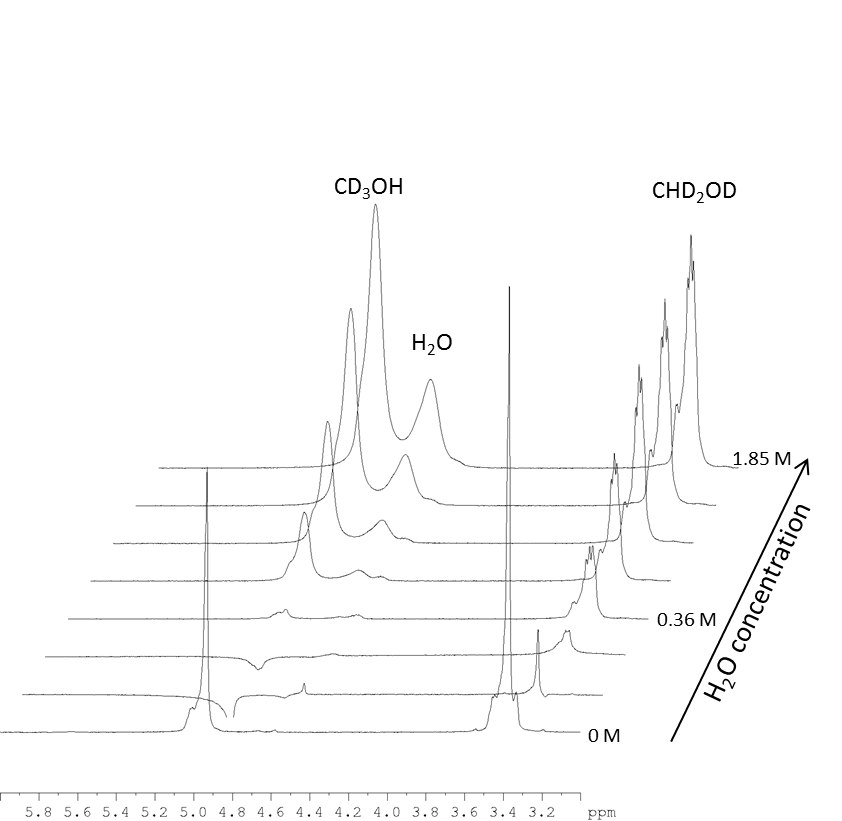


# Figure S4. Spectroscopic examination of ^1^H NMR signals of residual CD_3_OH, CHD_2_OD and H_2_O seen under SABRE.

Figure S4 shows that the level of SABRE induced signal gain in the residual CD_3_OH, CHD_2_OD and H_2_O resonances is concentration dependent. Figure S4 portrays this as a series of relative signal enhancement levels.

# Normalised ^1^H NMR signal enhancement levels, measured in the presence of water (M) and additional CD_3_OH after transfer at 70 G.

Figure S5 shows the decrease in ^1^H NMR signal enhancements seen for the protons in indazole and NCMe with increasing amounts of H_2_O (a) and CD_3_OH (b). The initial solution was 6.5 mM in **1a**, and contained 7-equivalents of indazole and 3 equivalents of NCMe relative to iridium.


**Figure S5.** ^1^H NMR signal enhancement values measured for indazole , NCMe, H_2_O and CD_3_OH as a function of H_2_O (see Figure 5a) and CD_3_OH concentration (see Figure 5b).

# SABRE derived ^13^C NMR signal enhancements seen for indazole as a function of the polarization transfer field.

**Table S2.** ^13^C NMR signal enhancement levels seen for indazole and NCMe as a function of the polarization transfer field strength with precatalysts **1a** and **1b**. The solutions used employed 6.5mM [Ir], and 10 and 3 equivalents of indazole and NCMe respectively.

| Field | **SIMes** | | | **IMes** | | |
| --- | --- | --- | --- | --- | --- | --- |
|  | **NCMe** | **A** | **B** | **NCMe** | **A** | **B** |
| 0G |  | 34 |  |  | 39 |  |
| 10G |  | 28 |  |  | 31 |  |
| 20G | 30 |  |  | 33.6 |  |  |
| 30G |  | 84 |  |  | 94 |  |
| 40G |  | 126 |  |  | 132 |  |
| 50G |  | 36 | 53 |  | 40 | 58 |
| 60G |  | 29 | 48 |  | 32 | 54 |
| 70G |  | 15 |  |  | 37 |  |
| 80G |  | 24 | 40 |  | 26 | 44 |
| 90G |  | 26 | 39 |  | 29 | 44 |
| 100G |  | 76 |  |  | 85 | 25 |
| 110G |  | 69 |  |  | 77 |  |
| 120G |  | 31 |  |  | 35 |  |

**Table S3.**  ^13^C NMR signal enhancement values seen for indazole and NCMe as a function of the polarization transfer field strength with the *d*_22_-IMes and *d*_22_-SIMes forms of **1a** and **1b**. The solutions used 6.5mM [Ir], and 10 and 3 equivalents of indazole and NCMe respectively.

| ^13^C | ***d*_22_-SIMes** | | | ***d*_22_-IMes** | | |
| --- | --- | --- | --- | --- | --- | --- |
|  | **NCMe** | **A** | **B** | **NCMe** | **A** | **B** |
| 0G |  | 26 |  |  | 22 |  |
| 10G | 24 | 25 |  |  | 15 |  |
| 20G | 33 | 29 |  | 15 | 16 |  |
| 30G | 27 | 51 |  | 14 | 42 |  |
| 40G |  | 53 | 26 |  | 23 |  |
| 50G |  | 23 | 27 |  | 14 | 23 |
| 60G |  | 23 | 28 |  | 10 | 15 |
| 70G |  |  |  |  |  |  |
| 80G |  |  | 32 |  |  | 23 |
| 90G |  |  |  |  | 16 | 13 |
| 100G |  | 46 |  |  | 63 |  |
| 110G |  | 39 |  |  | 35 |  |
| 120G |  |  |  |  | 11 |  |

# ^15^N NMR signal enhancement seen for NCMe.

We further note that the ^15^N NMR signal of unlabelled CH_3_CN is readily visible after transfer at ~0 G under for both the **1a** and **1b** precatalyst in the presents of indazole co-ligand**.**

**Figure S6:** ^15^N NMR signal enhancement seen for acetonitrile, with complex **1a** (below) and *d*_22_-**1a (**above) after transfer at ~0 G in a µ-metal shield. The solutions used 6.5mM [Ir], and 10 and 3 equivalents of indazole and NCMe respectively.

# Characterisation data for 5a.

**[Ir(H)_2_(imidazole)_3_(IMes)]Cl (5a)**: ^1^H NMR (400 MHz, MeOD, 294 K): δ -22.33 (2, 1H, hydrides), 2.02 (12H, C*H*_3_, IMes), 2.32 (6H, C*H*_3_, IMes), 6.08, 6.64 (2 1H, -C*H*=CH-, imidazole in *axial* position), 6.81 (4H, -C*H*=C*H*-, IMes), 6.84, 6.89 (4 1H, -C*H*=CH-, imidazole in *equatorial* position), 6.91 (2H, -C*H*=C*H*-, IMes), 6.97 (1H, N=C*H*-NH, imidazole in *axial* position), 6.99 (1H, N=C*H*-NH, imidazole in *equatorial* position). ^13^C NMR (400 MHz, 294 K): δ 17.4 (*C*H_3_, IMes), 19.6 (*C*H_3_, IMes), 115.3 (NH-*C*H=CH-, imidazole in *axial* position), 121.4, 127.9 (N*C*H=*C*HN, IMes), 121.9 (NH-*C*H=CH-, imidazole in *equatorial* position), 130.0 (N-*C*H=CH-, imidazole in *equatorial* position), 130.1 (N-*C*H=CH-, imidazole in *axial* position), 135.5, 137.6, 138.2 (-C=, IMes), 140.3 (N=*C*H-NH-, imidazole in *equatorial* position), 140.7 (N=*C*H-NH-, imidazole in *axial* position), 192.4 (N*C*N, IMes).

# Characterisation data for 5b.

**[Ir(H)_2_(imidazole)_3_(SIMes)]Cl (5b)**: ^1^H NMR (400 MHz, MeOD, 294 K): δ -22.12 (2, 1H, hydrides), 2.21 (12H, C*H*_3_, SIMes), 2.26 (6H, C*H*_3_, SIMes), 3.78 (4H, -C*H*_2_-C*H*_2_-, SIMes), 6.01, 6.64 (2H, -C*H*=CH-, imidazole in *axial* position), 6.75 (4H, -C*H*=, SIMes), 6.82, 6.88 (4H, -C*H*=CH-, imidazole in *equatorial* position), 6.89 (1H, N=C*H*-NH, imidazole in *axial* position), 6.95 (1H, N=C*H*-NH, imidazole in *equatorial* position). ^13^C NMR (400 MHz, 294 K): δ 17.5 (*C*H_3_, SIMes), 19.6 (*C*H_3_, SIMes), 115.2 (NH-*C*H=CH-, imidazole in *axial* position), 115.2 (NH-*C*H=CH-, imidazole in *equatorial* position), 130.0 (N-*C*H=CH-, imidazole in *equatorial* position), 130.1 (N-*C*H=CH-, imidazole in *axial* position), 128.2, 139.0, 140.3 (-C=, SIMes), 138.9 (N=*C*H-NH-, imidazole in *equatorial* position), 140.4 (N=*C*H-NH-, imidazole in *axial* position), 181.1 (N*C*N, SIMes).

# Relaxed and polarized ^1^H-NMR spectra in methanol-*d*_4_ of imidazole.


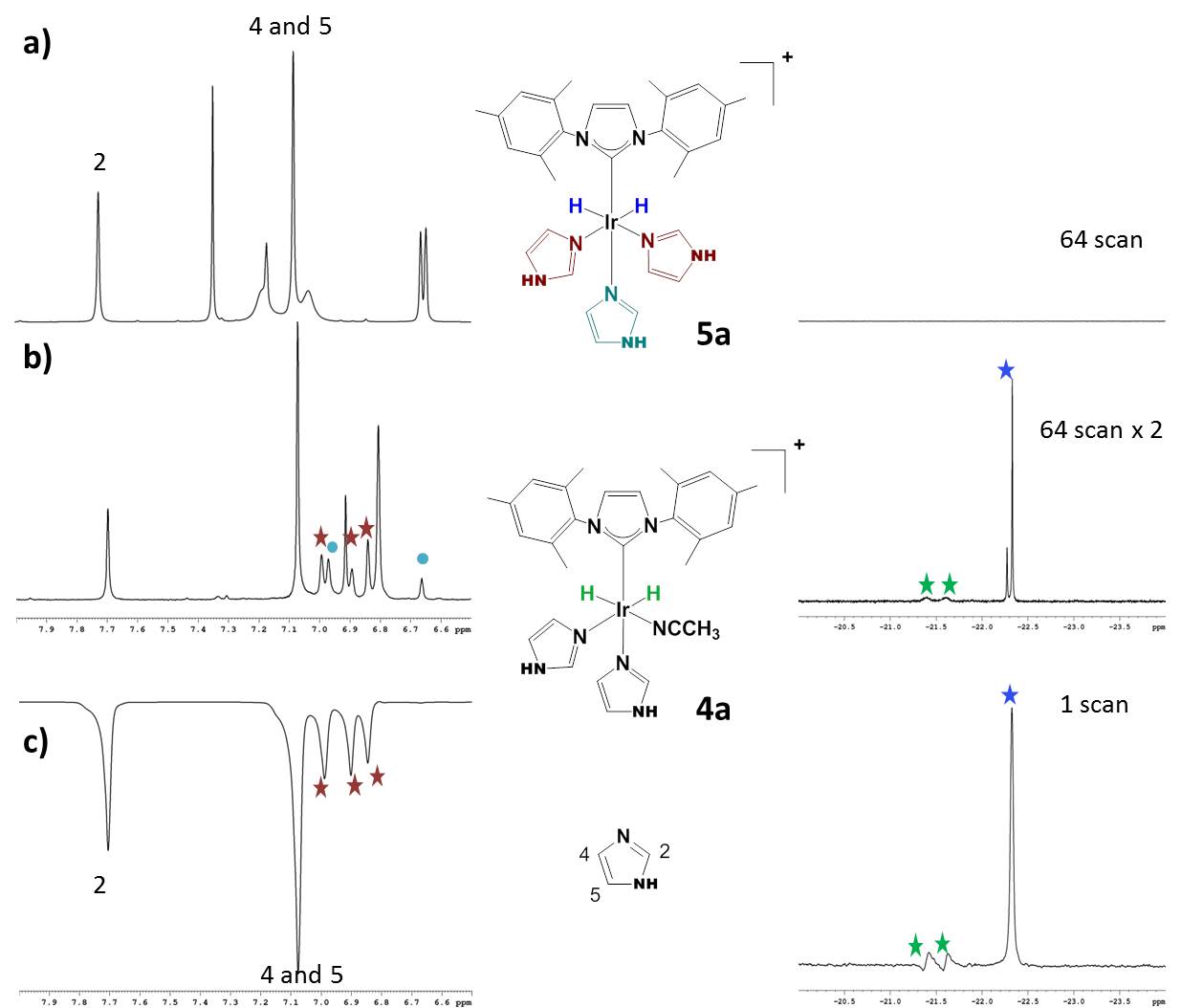


♦

**Figure S7:** Panes (a), (b) and (c) show the aromatic and hydride regions of the corresponding ^1^H-NMR spectra in methanol-*d*_4_ with imidazole.

Figure S7 contains panes (a), (b) and (c) that show the aromatic and hydride regions of a series of ^1^H-NMR spectra that were obtained in methanol-*d*_4_ with imidazole and **1a**. The NMR trace in pane (a) corresponds to that obtained before the addition of H_2_, and signals for H-2 through H-5 of imidazole are indicated. The addition of H_2_ changes this response as **4a** and **5a** form with the new resonances for the coordinated imidazole ligands being indicated with the * and ● labels (equatorial and axial ligands respectively). The hydride region (right) reveals that **5a** dominates, and that ^2^H label incorporation proceeds to form **5a**-HD (♦) in addition to **5a**-HH (*).

When this reaction is completed with *p*-H_2_, and observed by ^1^H NMR spectroscopy, the hydride ligand signals of **4a** exhibit antiphase character due to the PHIP effect in the corresponding ^1^H NMR spectra (Figure S7c). The SABRE enhanced signals for imidazole are illustrated in the left pane.

# Polarization transfer field profile for the SABRE enhancement seen for the ^1^H NMR signals of imidazole as achieved using the precataysts [IrCl(IMes)(COD)] and [IrCl(*d*_22_-IMes)(COD)].

Sample parameters used to collect the data of Figure S8: 6.5 mM [Ir], 10 equivalents of imidazole, 3 equivalents of NCMe. Labelling according to the inset structure. Signals gains per proton.

b)

# Figure S8. Polarization transfer field profile for the SABRE enhancement of the ^1^H NMR signals of imidazole achieved using the precatayst: a) [IrCl(IMes)(COD)] and b) [IrCl(*d*_22_-IMes)(COD)].

# Polarization transfer field profile for the SABRE enhancement seen for the ^1^H NMR signals of imidazole achieved using the precataysts [IrCl(SIMes)(COD)] and [IrCl(*d*_22_-SIMes)(COD)].

Sample parameters used to collect the data of Figure S9, 6.5 mM [Ir], 10 equivalents of imidazole, 3 equivalents of NCMe. Labelling according to the inset structure. Signal gains per proton illustrated.

a)

b)

# Figure S9. Polarization transfer field profile for the SABRE enhancement values seen for the ^1^H NMR signals of imidazole achieved using the precataysts: a) [IrCl(SIMes)(COD)] and b) [IrCl(*d*_22_-SIMes)(COD)].

# Polarization transfer field profile for the SABRE enhancement values determined for the ^1^H NMR signals of imidazole using the precataysts [IrCl(*d*_22_-IMes)(COD)] and [IrCl(*d*_22_-SIMes)(COD)] under acidic conditions.

Sample parameters used to collect the data of Figure S10, 6.5 mM [Ir], 10 equivalents of imidazole, 3 equivalents of NCMe and 5 mM HCl_aq_. Labelling according to the inset structure. Signal gains per proton illustrated.

**
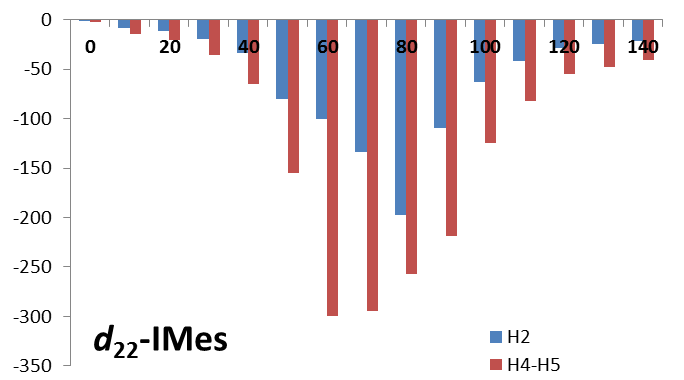
**a)

b)


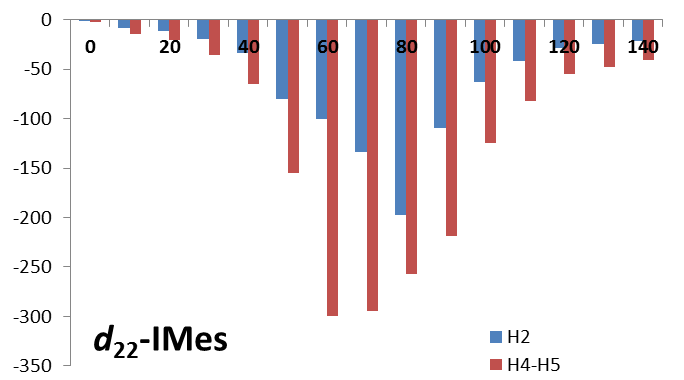


# Figure S10. Polarization transfer field profile for the SABRE enhancement values seen for the ^1^H NMR signals of imidazole achieved using the precataysts: a) [IrCl(*d*_22_-IMes)(COD)] and b) [IrCl(*d*_22_-SIMes)(COD)].

# Polarization transfer field profile for the acetonitrile ^1^H NMR signal response in solution with imidazole and [IrCl(IMes)(COD)], [IrCl(*d*_22_-IMes)(COD)], [IrCl(SIMes)(COD)] or [IrCl(*d*_22_-SIMes)(COD)].

Polarisation transfer field plots showing how the ^1^H NMR signal intensity gains seen for NCMe vary as a function of catalyst. 6.5 mM [Ir], 10 equivalents of imidazole and 3 equivalents of NCMe.


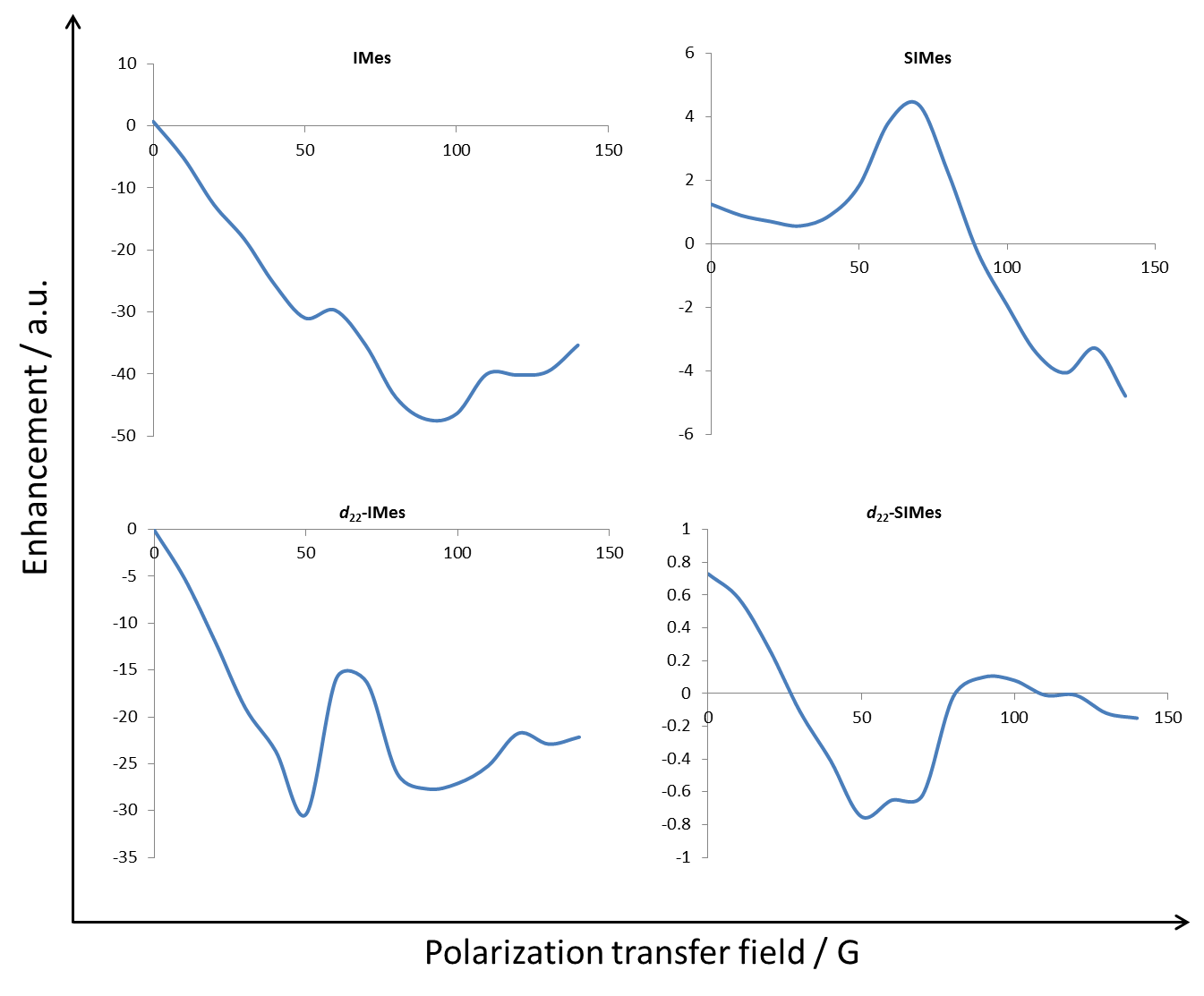


**Figure S11:** Polarisation transfer field plot showing how the ^1^H NMR signal intensity gains seen for NCMe change with catalyst, the catalysts used are labelled in the figures.

As described in the main text of the paper, polarization transfer into the protons of acetonitrile with **5a** is dramatically reduced.

# SABRE derived ^13^C NMR signal enhancement data for imidazole.

**Table S4** ^13^C NMR signal enhancement values seen for imidazole as a function of the polarization transfer field strength, with complexes **1a** and **1b**. Conditions: 6.5mM [Ir], 10 equivalents imidazole, 3 equivalents NCMe and 5.2 mM HCl.

|  | **SIMes** | | | | **IMes** | | | |
| --- | --- | --- | --- | --- | --- | --- | --- | --- |
| **^13^C** |  | | **+ HCl** | |  | | **+ HCl** | |
|  | **A** | **B** | **A** | **B** | **A** | **B** | **A** | **B** |
| 0G | 29 | 24 | 29 | 26 | 7 | 8 | 8 | 8 |
| 10G | 108 | 52 | 71 | 266 | 9 | 7 | 9 | 27 |
| 20G | 183 | 68 | 153 | 366 | 20 | 9 | 19 | 35 |
| 30G | 306 | 75 | 270 | 451 | 39 | 7 | 37 | 40 |
| 40G | 343 | 81 | 304 | 424 | 38 | 7 | 37 | 31 |
| 50G | 262 | 54 | 170 | 247 | 10 | 8 | 32 | 28 |
| 60G | 82 | 40 | 78 | 160 | 12 | 9 | 10 | 11 |
| 70G | 88 | 22 | 92 | 134 | 5 | 7 | 10 | 13 |
| 80G | 258 | 35 | 228 | 170 | 49 | 8 | 24 | 5 |
| 90G | 404 | 48 | 365 | 298 | 61 | 7 | 59 | 29 |
| 100G | 446 | 86 | 331 | 439 | 43 | 8 | 11 | 39 |
| 110G | 246 | 89 | 202 | 489 | 17 | 7 | 12 | 29 |
| 120G | 129 | 83 | 124 | 351 | 10 | 9 | 8 | 25 |

**Table S5**  ^13^C NMR signal enhancement values seen for imidazole as a function of the polarization transfer field strength with complexes **1a**-*d*_22_ and **1b**-*d*_22_. Conditions: 6.5mM [Ir], 10 equivalents imidazole, 3 equivalents NCMe and 5.2 mM HCl.

|  | ***d*_22_-SIMes** | | | | ***d*_22_-IMes** | | | | |
| --- | --- | --- | --- | --- | --- | --- | --- | --- | --- |
| **^13^C** |  | | **+ HCl** | |  | | | **+ HCl** | |
|  | **A** | **B** | **A** | **B** | **A** | **B** | | **A** | **B** |
| 0G | 29 |  | 35 | 31 | 31 | - | | 28 | 30 |
| 10G | 76 |  | 74 | 190 | 26 |  | | 28 | 100 |
| 20G | 120 |  | 108 | 217 | 24 |  | | 28 | 88 |
| 30G | 200 |  | 221 | 323 | 47 |  | | 52 | 138 |
| 40G | 235 |  | 214 | 228 | 59 |  | | 112 | 149 |
| 50G | 164 |  | 130 | 125 | 59 |  | | 90 | 107 |
| 60G | 58 |  | 83 | 148 | 40 |  | | 41 | 93 |
| 70G | 31 |  | 43 | 57 | 39 |  | | 33 | 106 |
| 80G | 96 |  | 59 | 86 | 96 |  | | 144 | 66 |
| 90G | 232 |  | 129 | 146 | 243 |  | | 87 | 144 |
| 100G | 248 |  | 217 | 204 | 139 |  | | 61 | 137 |
| 110G | 274 |  | 158 | 225 | 57 |  | | 34 | 99 |
| 120G | 134 |  | 84 | 142 | 29 | |  | 28 | 85 |

# ^15^N NMR signal enhancement on imidazole

The ^15^N NMR signal of unlabelled CH_3_CN is visible at 239 ppm after transfer at 0 G, in a µ-metal shield with both **1a** and **1b** precatalysts in the presence of the imidazole**.** Upon adding HCl, a strongly enhanced signal at 200 ppm for imidazole became visible.


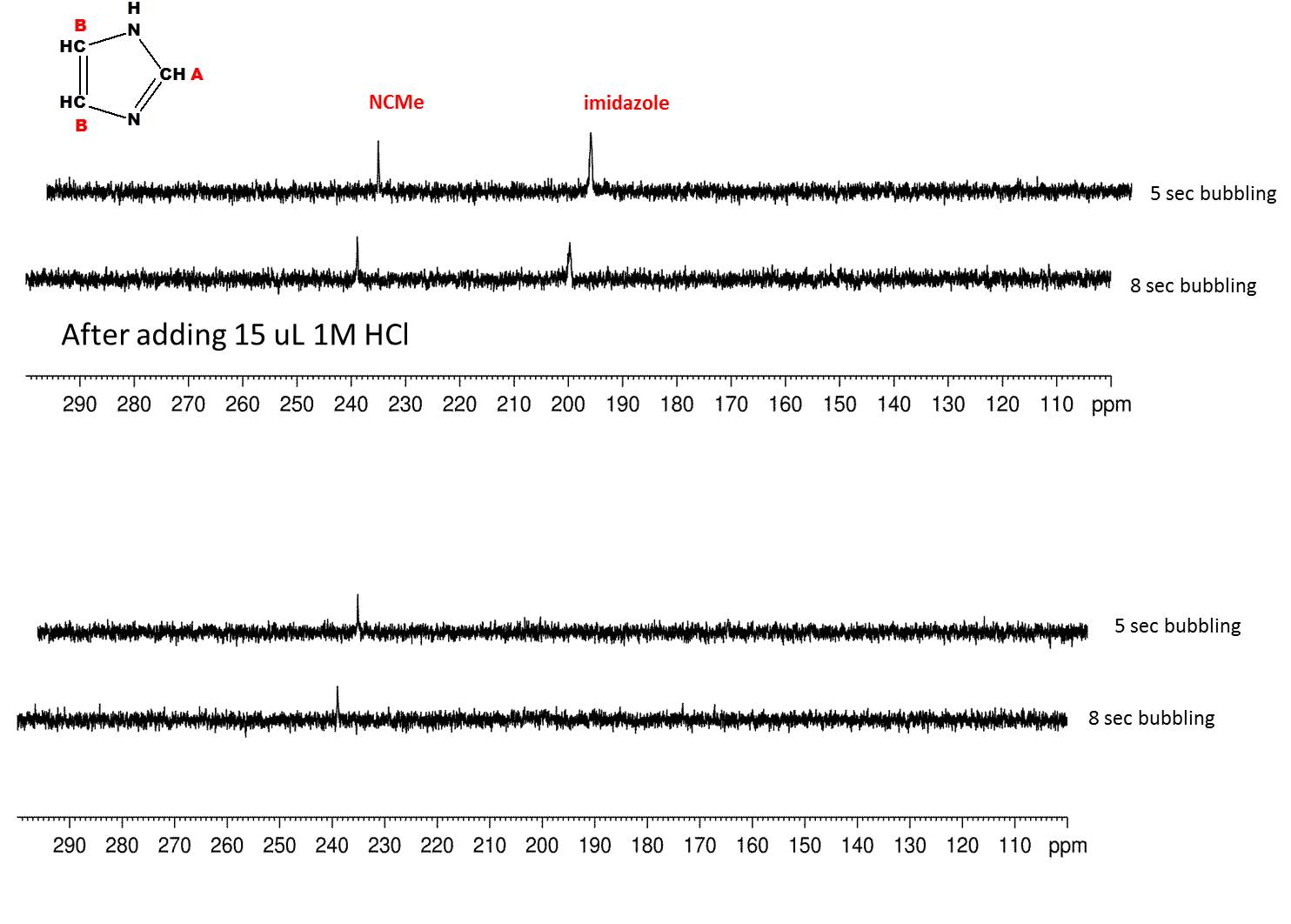


**Figure S12.** ^15^N NMR signals detected with precatalyst **1a** after transfer at ~0 G before and after adding 5.2 mM HCl to the system. Conditions: 6.5mM [Ir], 10 equivalents imidazole and 3 equivalents NCMe.

# References

[1] O. Torres, M. Martin and E. Sola, *Organometallics* **2009**, *28*, 863-870.

[2] M. Fekete, O. Bayfield, S. B. Duckett, S. Hart, R. E. Mewis, N. Pridmore, P. J. Rayner and A. Whitwood, *Inorganic Chemistry* **2013**, *52*, 13453-13461.

[3] D. Blazina, S. B. Duckett, J. P. Dunne and C. Godard, *Dalton Transactions* **2004**, 2601-2609.

[4] R. E. Mewis, K. D. Atkinson, M. J. Cowley, S. B. Duckett, G. G. R. Green, R. A. Green, L. A. R. Highton, D. Kilgour, L. S. Lloyd, J. A. B. Lohman and D. C. Williamson, *Magnetic Resonance in Chemistry* **2014**, *52*, 358-369.

[5] E. M. Leitao, S. R. Dubberley, W. E. Piers, Q. Wu and R. McDonald, *Chemistry – A European Journal* **2008**, *14*, 11565-11572.
